# The study of the *transformer* gene from *Bactrocera dorsalis* and *B. correcta* with putative core promoter regions

**DOI:** 10.1186/s12863-016-0342-0

**Published:** 2016-02-01

**Authors:** Kamoltip Laohakieat, Nidchaya Aketarawong, Siriwan Isasawin, Siripong Thitamadee, Sujinda Thanaphum

**Affiliations:** Department of Biotechnology, Faculty of Science, Mahidol University, Rama VI Road, Bangkok, 10400 Thailand

**Keywords:** Oriental fruit fly, Guava fruit fly, *Tra* core promoter, Alternative splicing, Genetic sexing strain

## Abstract

**Background:**

The *transformer* (*tra*) is a sex determining switch in different orders of insects, including Diptera, as in the family Tephritidae. The lifelong autoregulatory loop of *tra* female-specific splicing can be reset by the intervention of male-specific primary signals (*M* factor). In early development, the functional female and truncated male TRA proteins relay the sexual fates to the alternative splicing of a bisexual switch gene, *doublesex* (*dsx*) cascading the sexual differentiation processes. *Bactrocera dorsalis* (Hendel) and *Bactrocera correcta* (Bezzi) are among the *Bactrocera* model worldwide key pests. Area-wide integrated pest management using the male-only Sterile Insect Technique (SIT) relying on genetic sexing systems is effective in control programs. We undertook the molecular characterization and comparative studies of the *tra* orthologues in the *Bactrocera* species, including the Salaya1 genetic sexing strain (GSS).

**Results:**

RT-PCR revealed that *B. dorsalis tra* (*Bdtra*) and *B. correcta tra* (*Bctra*) transcripts contained conservation of both constitutive exons and male-specific exons as in other *Bactrocera*. However, new *Bdtra* male-specific exons were retained, diversifying the pattern of the male-specifically spliced transcripts. The coding sequences of *tra* were highly conserved in *Bactrocera* (86–95 %) but less so among related genera (61–65 %) within the same Tephritidae family. A conservation of deduced amino acid sequences (18 residues), called the TEP region, was identified to be distinctive among tephritids. The 5’ regulatory sequence containing many structural characteristics of the putative core promoter was discovered in *B. correcta.* The expression patterns of *Bdtra* and *Bctra* were sex-specifically spliced and the signals relayed to the *dsx* genes in the adult wild-types. However, the coexistence of male- and female-specifically spliced transcripts (980 and 626 bp, respectively) of the *B. dorsalis* wild-type strain was found in the Salaya1 GSS adult males. The *Bdtra* RNA interference masculinized the XX karyotype females into pseudomales, but their testes were mostly not well developed.

**Conclusions:**

*Bdtra* and *Bctra* have sex-specific splicing, similar to *Bactroceras*, *Ceratitis capitata* (Wiedemann), and *Anastrephas*. A newly identified TEP region is proposed in tephritids. A putative core promoter has been discovered in *Bctra*.

**Electronic supplementary material:**

The online version of this article (doi:10.1186/s12863-016-0342-0) contains supplementary material, which is available to authorized users.

## Background

Eukaryotic sexual reproduction is an important biological process for the continuity of life. Meiosis provides diverse gametes which can be reunited through sexual selection and fertilization. The progeny, endowed with a suitable genome, are naturally selected before the next round of mating. The male and female developmental dimorphism is fundamental for the existence of sexual characteristic [1].

The sex determination of insects is one of the most well-known system [2, 3]. There are several mechanisms that determine the sexual fate among insect models [3, 4]. The primary signals convey genetic instruction to the sex determination switches in order to designate and sustain sexual identity during development and throughout an organism’s life [3, 4]. In very early embryonic development (approximately before the blastoderm cellularization), an upstream regulator is usually required to be established as a stable genetic switch that serves as a device memory for sex-specific cell fate [5–15].

In *Drosophila melanogaster*, the primary signal is composed of proteins encoded by a group of genes linked to the X-chromosome, called X-linked signal elements (XSE) [16, 17]. Their encoded proteins interact with the other group of proteins encoded by autosomally localized genes to generate sex-specific dosages (XX/AA or XY/AA) [17, 18]. These dosages regulate the transcription of *Sex-lethal* (*Sxl*), upstream regulator, which is also an X-linked gene that presents in both sexes. However, only the XX/AA female dosage can activate the early female-specific promoter of the *Sxl* gene. This early female-specific SXL protein is a sex-specific splicing regulator of the later promoter *Sxl* pre-mRNA. The secondary SXL protein can autoregulate the female-specific splicing of its own pre-mRNA [17–20] and the subordinated control gene as *transformer* (*tra*) to maintain female development [21, 22]. In contrast, with males, the absence of early SXL protein leads to the production of male-specifically spliced *Sxl* transcripts which incorporate in-frame stop codons, resulting in the truncated male-specific SXL protein [19, 23].

In *Ceratitis capitata* (Wiedemann), a tephritid fruit fly, the primary signal is an uncharacterized *male-determiner M* (*M* factor) but was only mapped on a Y-chromosome [24]. The *M* factor was proposed to regulate the *tra* gene, in *C. capitata* [6, 7, 25]. It was postulated that the *M* factor inhibited the maternal TRA protein, which is required for female-specific splicing of *tra* pre-mRNA and results in a zygotic male-specifically spliced *tra* mRNA [6, 7, 25]. The *tra* gene is an upstream regulator of *dsx* and *fru* genes in *C. capitata* because the embryonic RNAi against *tra* and/or *tra-2* experiments led to a stable splicing pattern change in *tra*, *dsx*, and *fru* pre-mRNAs only in XX individuals [7, 25]. In the other tephritid fruit flies, such as the *Bactrocera* species and *Anastrepha* species, the *tra* genes show similar molecular organization and expression of hierarchy patterns to those found in *C. capitata* [8, 26–29]. These results suggest that the *tra* orthologues may be the upstream regulators under the *M* factor control. The alternative sex-specific splicing of the *tra* gene is under the control of its own autoregulatory loop [6–8, 25–31]. This decision is proposed to be initiated by the signal from the maternal TRA protein, where the *M* factor is absent in XX females [6–8, 25]. In *Drosophila*, the functional TRA protein co-operates with the constitutively expressed Transformer-2 (TRA-2) protein, and the complex acts as a splicing regulator [32, 33]. However, in *C. capitata*, the functional analysis of TRA/TRA-2 complex was indirectly inferred from the RNAi against *Cctra-2* effecting *tra* splicing as well as *dsx* and *fru* splicing in XX individuals only [25]. Since the functional analysis of the TRA/TRA-2 complex has been experimentally proven only in *D. melanogaster*, it was postulated that in *C. capitata* the TRA/TRA-2 complex may bind to the putative TRA/TRA-2 binding sites located on the male-specific exons and their flanking introns in order to block the strong splice site of male-specific exons. Thereafter, the other SR proteins and spliceosome machinery may be recruited, and the male-specific exons are skipped, resulting in female-specific splicing and the production of a functional TRA protein. On the other hand, in males where the *M* factor is present, this entire situation may be turned off. Without TRA/TRA-2 regulation, the splicing may be followed by the default which contains in-frame stop codons on male-specific exons and leads to the production of a non-functional truncated TRA protein [6–8, 25–31]. The functional CcTRA proteins may be the sex-specific splicing regulators [7]. They may not only maintain their autoregulatory loop but may regulate the alternative sex-specific splicing of a highly conserved bottom global effector in the sex determination pathway, the so-called *doublesex* (*dsx*) gene [34]. The sex determining message carried by this DSX protein can execute sexual differentiation processes in a very broad scheme.

The male- and female-specific DSX (DSX^M^ and DSX^F^) proteins play transcription factor roles to regulate the downstream genes involved in the sexual differentiation [32, 34]. Most of the mechanism underlining the regulatory roles of the TRA/TRA-2 complex for *dsx* sex-specifically spliced transcripts was only experimentally proven in *D. melanogaster* [32–36] while much indirect evidence was gathered from the other tephritid species [37–44]. The presence of functional TRA and TRA-2 proteins acts as a splicing activator that binds to the *dsx* repeat element (*dsx* RE) on the female-specific exon. This binding makes a nearby *cis*-purine rich element (*dsx* PRE) become a stronger splicing acceptor that facilitates female-specific splicing resulting in the DSX^F^ protein. In contrast, the presence of truncated TRA cancels the strength and potential of this splicing acceptor in males due to no TRA/TRA-2 binding [32–36]. Therefore, the male-specifically spliced *dsx* transcript contains male-specific exons. This leads to the production of the DSX^M^ protein. The highly conserved *dsx* genes were also cloned and characterized in many tephritid fruit fly models [37–42] including *Bactrocera dorsalis* (Hendel) [43, 44] and *Bactrocera correcta* (Bezzi) [44]. The sex determination pathway would be better understood if *tra* [7, 8, 26–29] and *tra-2* [8, 25, 28–30] counterparts were also characterized. Moreover, it would be advantageous to carry out comparative studies of *tra* and *dsx* relationships within the tephritid group to refine the model of sex determination. The role of *tra* in the sex determination pathway and its related practical applications in pest control programs can be further understood and realized when molecular comparisons are carried out in model organisms belonging to a highly diverse agriculture pest genus such as *Bactrocera*. The oriental fruit fly (*B. dorsalis*) and guava fruit fly (*B. correcta*) belong to the *Bactrocera* genus, are economically important pests, and are therefore considered targets for control programs [45].

In this study, we isolated and characterized *tra* genes from *B. dorsalis* and *B. correcta* as well as their putative core promoters. After the full-length cDNA of adult males and females were obtained, RT-PCR was performed with appropriate primers to elucidate the alternative sex-specific splicing in male and female transcripts. The structure and amino acid sequences of both genes were highly conserved among the *Bactrocera* species, but less so in other tephritid fruit flies. Expression analyses of *tra* genes (the upstream regulators) were also studied in relation to the *dsx* genes (the bottom effector) in the wild-type strain (*B. dorsalis*) and the Salaya1 genetic sexing-mass-reared strain (*B. dorsalis*). Moreover, the *tra* RNAi experiment was carried out for the functional analysis of masculinization using the brown and white pupal sexual dimorphism of the Salaya1 strain as a sexual karyotype identifier. The results demonstrated the functions of the *tra* gene were highly conserved in the sex determination. In addition, the putative core promoter regions of *tra* genes in *B. dorsalis* and *B. correcta* represented the initial discovery of such within the tephritid species. The newly isolated speculative core promoter regions of *tra* genes from *Bactrocera zonata* (Saunders), *Bactrocera tryoni* (Froggatt), *Bactrocera carambolae* Drew & Hancock (this work); the previously isolated *Bactrocera oleae* (Gmelin) [26]; and the *dsx* putative core promoters from *B. dorsalis* and *B. correcta* [44] were also uncovered. Several molecular features of *tra* orthologues were compared across *Bactrocera* species and their related genera to untangle the sex determination mechanism, suggesting further practical genetic tools.

## Results and discussion

### Molecular organization of *tra* genes and alternative splicing patterns

**Fig. 1:**
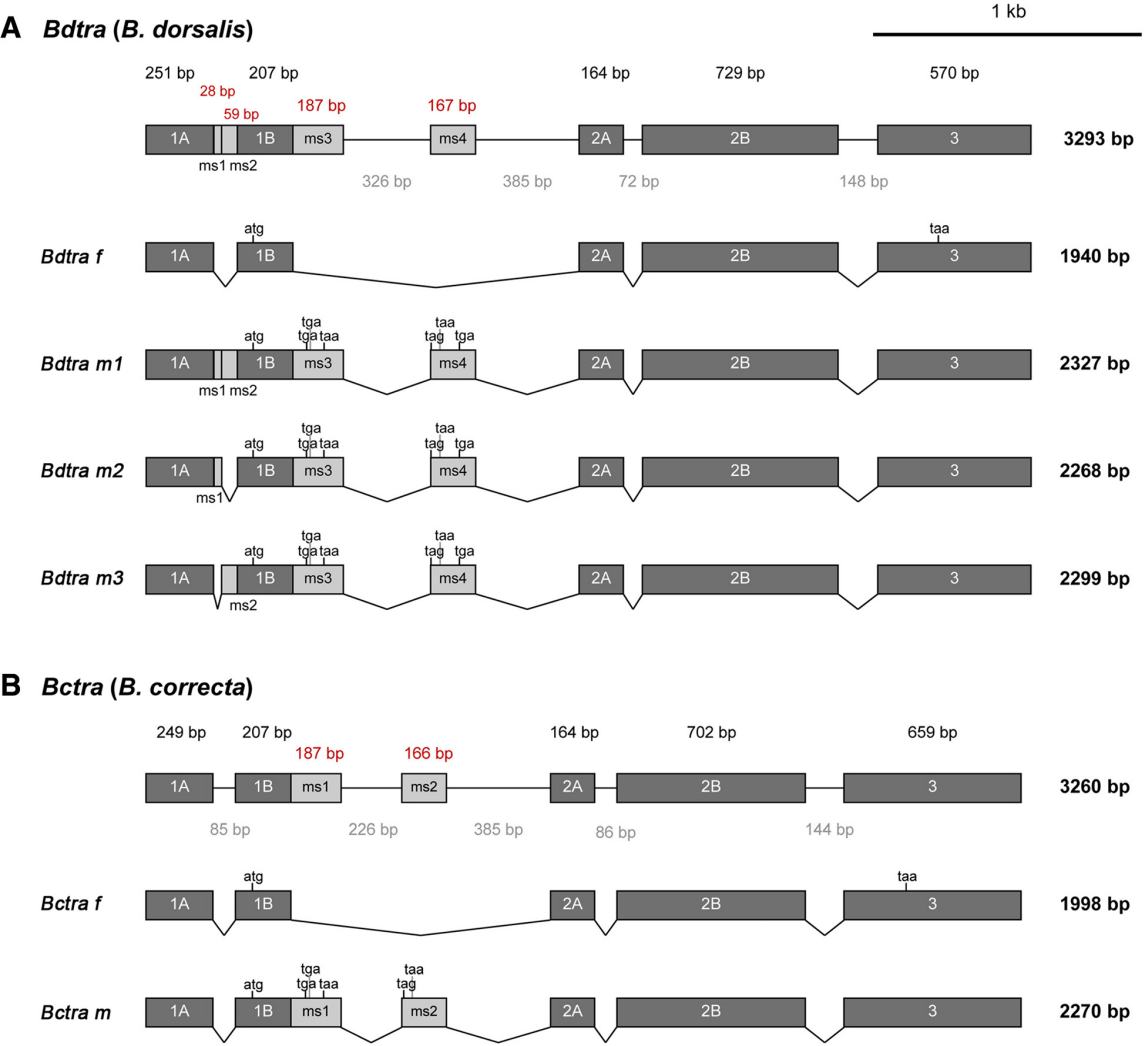
Molecular organization and sex-specific transcript of *tra* genes from *B. dorsalis* (**a**) and *B. correcta* (**b**). Both genes contain five common exons (*dark gray boxes*); however, there are four and two male-specific (ms) exons (*light gray boxes*) in *Bdtra* and *Bctra*, respectively. Introns are lines connecting exon boxes. The start codon (atg) is indicated in the common exon 1B while the conceptually alternative stop codons are either located in the downstream male-specific exons or in common exon 3 for the female transcript.

The molecular structure of the *B. dorsalis tra* (*Bdtra*) gene contained five common exons corresponding to exons 1A, 1B, 2A, 2B, and 3, as in other *Bactrocera* species [8, 26, 28, 29]. The common exons 1A and 1B of *Bdtra* were homologous to exon 1 of *C. capitata tra* (*Cctra*) [7] and *Anastrepha obliqua* (Macquart) *tra* (*Aotra*) [27] genes. Two similar female transcripts, *Bdtra f1* [GenBank: KU254111] and *Bdtra f2* [GenBank: KU254112] were approximately 1.9 kb long but different in the length of a 51 bp indel at the 3’UTR (not shown). The open reading frame (ORF) of female transcripts was 1266 bp encoding for a predicted polypeptide of 422 amino acids (Fig. 1a). Three male transcripts, *Bdtra m1* [GenBank: KU254113], *Bdtra m2* [GenBank: KU254114], and *Bdtra m3* [GenBank: KU254115] were approximately 2.3 kb long. These transcripts contained two conserved male-specific exons (ms3 and ms4) inserted between the common exons 1B and 2A as also appeared in the other *Bactrocera* species [8, 26, 28, 29]. The in-frame stop codons in ms3 and ms4 conceptually lead to the production of a truncated TRA protein with 66 amino acids. Two novel male-specific exons (ms1 and ms2) between the constitutive exons 1A and 1B were found. These exons were either being alternatively spliced or coexisted in combination, resulting in three possible male-specific transcripts (Fig. 1a). The *Bdtra m1*, *Bdtra m2*, and *Bdtra m3* transcripts had a combination of ms1 and ms2 (87 bp), only ms1 (28 bp), and only ms2 (59 bp), respectively (Fig. 1a). In several *Bactrocera*, the intron between common exons 1A and 1B is constitutively spliced out [8, 28, 29]. Notably, the same intron is, however, observed to be specifically retained in *B. oleae* [26] and *B. dorsalis* (this work) males. Unlike the case of *B. oleae*, there are three polymorphic isoforms (*Bdtra m1* to *m3*). There are no recognizable TRA/TRA-2 binding sites, RBP1 (RNA-binding protein 1) binding sites, and TRA-2 ISS (intronic splicing suppressor) sequences around these newly discovered male-specific exons. This suggests that the diversification of male-specific exons is an active process in *B. dorsalis*. This seems to be made possible without the involvement of splicing regulation such as TRA/TRA-2. The evolution of TRA/TRA-2 may not be involved in the early steps of sex-specific splicing in the sex determination pathway. The selection of the male-specific splicing in these regions might be relaxed because it entirely involves the 5’ UTR [46]. In addition to this work, an intron between two common male-specific exons (ms3 and ms4) among other *Bactrocera* is totally retained with no obvious relationship to the TRA/TRA-2 binding site in *B. dorsalis* males (China population, [28, 29]). This fact also supports that the male-specific exon is evolving in *B. dorsalis*.

In *B. correcta*, the *tra* (*Bctra*) gene was approximately 2 and 2.3 kb long in female- and male-transcripts, respectively [GenBank: KU254116 and KU254117]. There was also conservation of the five common exons and the two male-specific exons (Fig. 1b). However, the novel male-specific exons found in the *Bdtra* transcripts were not evident. The ORF of the female-specific transcript was 1239 bp and encoded for 413 amino acids. However, the male TRA protein appeared truncated because there were in-frame stop codons located in the male-specific exons.

The male-specific exons of *tra* genes in tephritid fruit flies located between the common exons 1 and 2 in *C. capitata* [7] and *Anastrepha* species [27] or 1B and 2A in *Bactrocera* species [8, 26, 28, 29] contained at least three in-frame stop codons leading to the production of a non-functional TRA polypeptide. Moreover, these male-specific exons and their flanking introns had the putative splicing regulatory elements. These regions of *Bdtra* and *Bctra* were analyzed and then compared to the others: *Bactrocera* species [8, 26, 28, 29], *Anastrepha* species [27], *C. capitata* [7], and *D. melanogaster* [32] (Additional file 1: Figure S1 and Additional file 2: Table S1). Four putative regulatory elements (i.e., TRA/TRA-2, RBP1 binding sites, TRA-2 ISS sequences, and purine-rich elements) were observed [47, 48]. The two male-specific exons found between the two common exons 1B and 2A are also conserved in all *Bactrocera* species, as in the other tephritids. In this case, many of those putative regulatory elements were found to be clustered [7, 26, 27]. Future functional studies of these TRA/TRA-2 binding sites are required because there is only information regarding *dsx* and *fruitless* (*fru*) sex-specifically spliced genes from *D. melanogaster* [33, 36, 49] at present.

### Sex-specific splicing analysis

**Fig. 2:**
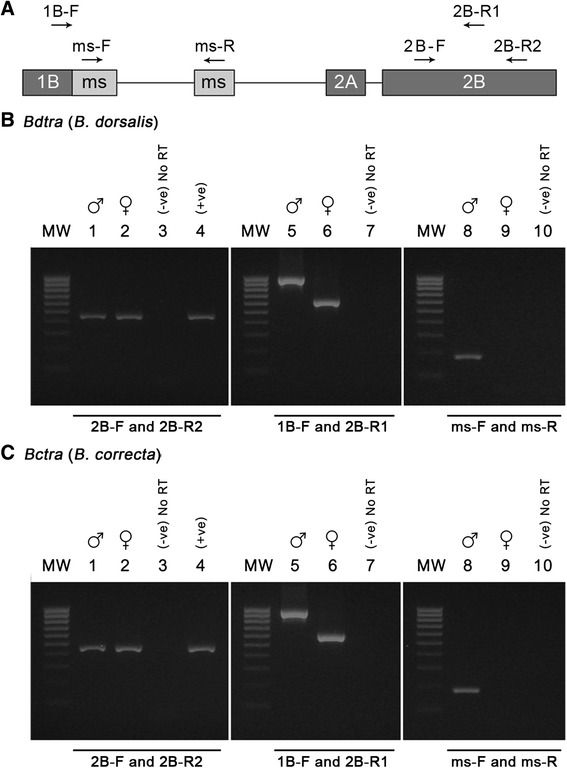
Detection of sex-specific *Bdtra* and *Bctra* transcripts by RT-PCR analysis from wild-type strains. **a** The location of appropriate primers is indicated by arrows. The total RNA was separately prepared from adult male and female wild-type flies: *B. dorsalis* (**b**) and *B. correcta* (**c**). PCR was performed using primers 2B-F and 2B-R2 in lanes 1 – 4, primers 1B-F and 2B-R1 in lanes 5 – 7, and primers ms-F and ms-R in lanes 8 – 10. Positive control was performed using genomic DNA as a template ((+ve), lane 4). Lanes 3, 7, and 10 are without RT as a negative control (−ve). MW is the 100 base pair molecular weight DNA marker.

Three sets of RT-PCR were carried out in males and females of *B. dorsalis* and *B. correcta*. These results demonstrated that *Bdtra* and *Bctra* pre-mRNA were alternatively sex-specific spliced. The first set of primers, 2B-F – 2B-R2, was located on common exon 2B (Fig. 2a), and the amplified RT-PCR product was identical in cDNA derived from both sexes and genomic DNA for 458 bp of *B. dorsalis* and 455 bp of *B. correcta* (Fig. 2b and c). The second set of primers, 1B-F – 2B-R1, was located on common exons 1B and 2B, respectively, flanking the male-specific exons (Fig. 2a). The amplified products were different in males and females. The male amplified products of 980 bp for *Bdtra* and 951 bp for *Bctra* were greater than the female amplified products of 626 and 599 bp for *Bdtra* and *Bctra*, respectively (Fig. 2b and c). The third set of primers, ms-F – ms-R, was located on male-specific exons (Fig. 2a). A specific band of 184 bp for *Bdtra* and 183 bp for *Bctra* was detected only in males, as expected (Fig. 2b and c), supporting that male transcripts contain in addition male-specific exons. Therefore, males encoded non-functional truncated TRA peptides and only the female transcript produced functional TRA polypeptides. These have been commonly found in other tephritid fruit flies [6–8, 26–29].

### 5’ upstream regulatory sequences and putative core promoters

**Fig. 3:**
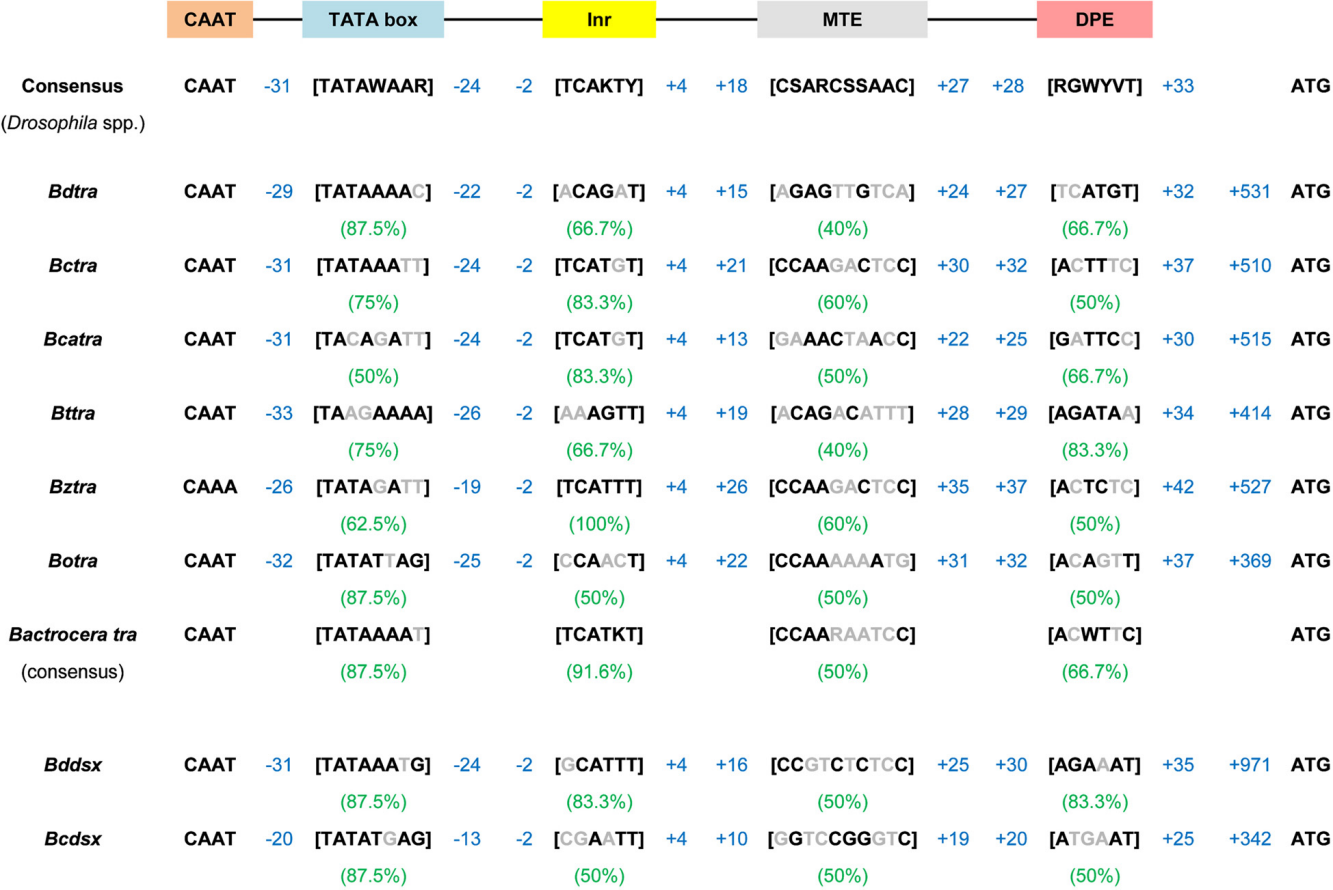
Schematic representation of hypothetical core promoters with conserved *cis*-regulatory motifs. The putative CAAT box, TATA box, Inr, MTE, and DPE elements are in linear arrangement with no scale (above). The *Drosophila* species-derived consensus sequences are in bold, in brackets, and below each box [50]. The relative positions of these elements are indicated by flanked blue numbers which are in reference to a hypothetical A (+1) initiator within the Inr motif. The number directly on the left of the start codon (ATG) also represents the relative distance from the same hypothetical A. The string of putative core promoter elements from each gene is arrayed in lines. The actual conserved nucleotide sequences are in bold and their overall identity (%) indicated underneath. *Bdtra*, *Bctra*, *Bztra*, *Bttra*, *Bcatra* (this work), and *Botra* [26] represent the putative 5’ upstream regulatory sequences of *tra* isolated from *B. dorsalis*, *B. correcta*, *B. zonata*, *B. tryoni*, *B. carambolae*, and *B. oleae*, respectively. *Bddsx* and *Bcdsx* represent the putative 5’ upstream regulatory sequences of *tra* isolated from *B. dorsalis* and *B. correcta*, respectively [44].

Appropriate inverse PCR primers were designed after the complete CDS of *Bdtra* and *Bctra* genes were obtained in order to amplify their 5’ upstream regulatory regions and putative core promoters. The length of the 5’UTRs (*Bdtra*, 341, 372, and 400 bp in males and 313 bp in females; *Bctra*, 311 bp in both sexes) was still an estimation based on the most upstream RT-PCR forward primer binding position carried out using the other reverse primer located within a coding region and total cDNA template experiments. A continual upstream series of RT-PCR primers was originally designed and tested from inverse PCR and genomic DNA templates, respectively. Normally, the 5’RACE is a conventional method to identify the 5’UTR length. In the previous work in the *B. dorsalis* China population (171 bp [28] and 117 bp [29]), the 5’UTR derived from such method was a few hundred bp shorter. The sequence upstream of the translation start site, including the 5’UTR of *Bdtra* and *Bctra*, encompassed 1439 bp [GenBank: KU254107] and 1496 bp [GenBank: KU254106], respectively. The 5’ upstream regulatory regions and putative core promoters of *Bdtra* and *Bctra* were AT rich regions (66.23 and 66.51 %, respectively). The upstream regions of both genes revealed to a certain degree the characteristics of putative core promoters for transcription by RNA polymerase II that consisted of a CAAT box, TATA box, Initiator (Inr), motif ten element (MTE), and downstream core promoter element (DPE) found in *D. melanogaster* [50, 51] (Fig. 3). The identification of these speculative *cis*-regulatory sequences was carried out by visual inspection for the most conserved putative TATA box and Inr sequences as per the *Drosophila* core promoter database [50–52] within the upstream regulatory regions.

**Fig. 4:**
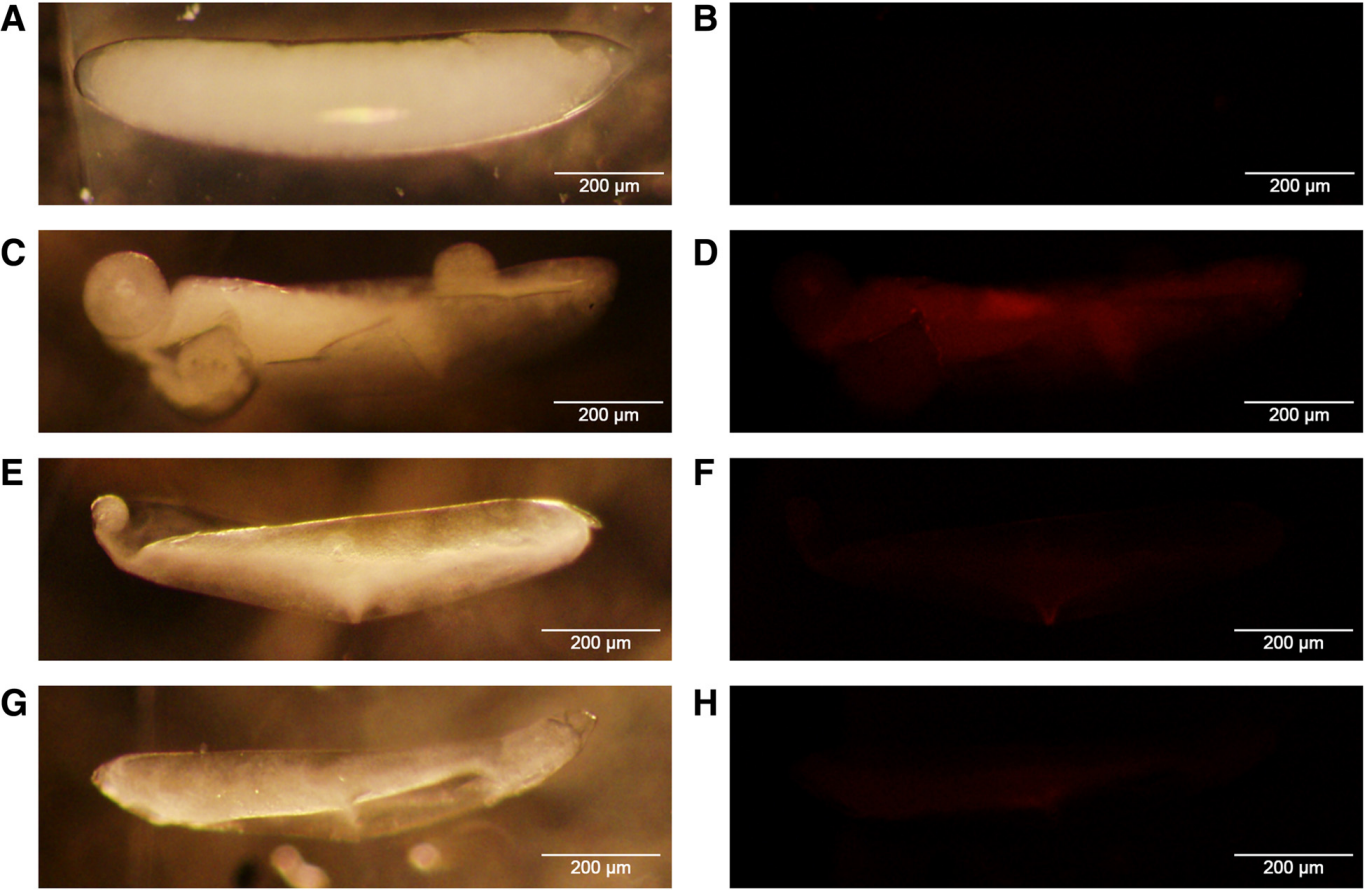
Transient expression analysis of the *Bctra* putative core promoter. The *DsRed* gene was used as a reporter. The #1215-*Bctra*-*DsRed* injected embryos were investigated. The left column figures (**a**, **c**, **e**, and **g**) are investigated under bright-field microscopy and the adjacent figures (**b**, **d**, **f**, and **h**) are the same embryos under epifluorescence. (**a**) and (**b**) represent an uninjected embryo. (**d**), (**f**), and (**h**) are the injected embryos at 31 hours after egg-laying and show red fluorescence.

The characteristics of the putative core promoter were observed in the *Bdtra* and *Bctra* that covered positions −29 to +32 and −31 through +37 in relation to an A (+1) in the Inr sequence, respectively (Fig. 3). The functional analysis of the 5’ upstream regulatory region and putative core promoter of *Bctra* gene was performed using the *DsRed* as a reporter gene with *SV40* poly A signal (#1215-*Bctra*-*DsRed* constructs). The *Bctra*-putative promoter sequence driving *DsRed* construct was created and microinjected into the early embryos of the *B. correcta* wild-type strain. The microinjected embryos (G_0_) representing developmental times (from 1 to 48 hours after egg laying (h AEL)) were screened for transient DsRed fluorescence and compared with the uninjected embryos. A consistently low level of DsRed fluorescence was detected after the 19–20 h AEL, with no fluorescence signal observed in the uninjected embryos. The brightest DsRed fluorescence was observed in embryos at 31 h AEL (Fig. 4). When this *Bctra*-putative promoter sequence driving *DsRed* construct was cross-species microinjected into the early embryos of *B. dorsalis* wild-type strain, no positive result was observed. This supports that the *tra* promoter may be species-specific although further, more intensive functional characterization will be needed to confirm this propensity. The evidence suggests the discovery of a *tra* putative core promoter in *B. correcta*. Moreover, the upstream regions of putative core promoters covering positions −909 through −278 of *Bdtra* and positions −987 through −306 of *Bctra* were 96-100 % identical to the predicted *B. dorsalis* uncharacterized transcript LOC105232903 gene [GenBank: XM_011214797.1] in head-to-head orientation.

The unique finding of this work is the identification of a region that can act as a *tra* promoter (*Bctra*). This region may be a core promoter region because it contains many conserved putative core promoter elements. For example, the putative TATA box and putative Inr sequence were 75 and 83.3 % identical, respectively, to their consensus sequences from the *Drosophila* core promoter database (Fig. 3) [50–52]. In addition, the location of the upstream “T” in the putative TATA box was exactly at the −31 position in relation to the “A” in the putative Inr consensus sequence, concurring with the TATA box location preference with regard to the Inr motif [50]. The other less conserved putative core promoter elements such as the MTE and DPE (60 and 50 % similarity, respectively) were also observed. Whether any of these putative core promoter elements are functional remains to be proven by further promoter analysis experiments.

The 5’ upstream regulatory sequences of *tra* genes were also amplified from *B. tryoni*, *B. carambolae*, and *B. zonata* using primers Tpro and 1B-1R specific to LOC105232903 and *tra* CDS, respectively (this work). The upstream sequence of the translation start site (ATG) of *Bttra* [GenBank: KU254110], *Bcatra* [GenBank: KU254109], and *Bztra* [GenBank: KU254108] encompassed 1126, 1271, and 1268 bp, respectively. These regions were also AT rich (67 %), similar to *B. dorsalis* and *B. correcta*. The putative core promoter motifs were observed in *B. tryoni*, *B. carambolae*, *B. zonata*, and *B. oleae* [26] in similar vicinities. Moreover, similar putative core promoters were also present in the previously characterized *dsx* genes of *B. dorsalis* and *B. correcta* [44] (Fig. 3). Again, promoter analysis experiments would be required to prove the detailed functions regarding these speculations.

### Conservation of *tra* genes and the newly recognized TRA protein region within tephritid fruit flies

The female functional TRA proteins and their encoded genes were compared across tephritid species and *D. melanogaster*. The identity at the nucleotide level of *tra* genes was analyzed among these species and then the conservation pattern was compared to the *dsx* genes (Table 1). The *tra* gene was highly conserved (86–95 and 98 % identical) within the genera of *Bactrocera* and *Anastrepha*, respectively. On the other hand, a lower level of similarity (61–65 % identity) was detected in the comparison among those genera and *Ceratitis* within the same family. The identity of *dsx* genes was relatively more conserved among the same related genera than the identity of *tra* genes (87–88 % versus 61–65 %) (Table 1) [8, 37–44]. The phylogram of *tra* ORF was reconstructed and the result supported a close and monophyletic relationship of the *Bactrocera* genus, separate from the other tephritid clades (Additional file 3: Figure S2).

**Table 1:** The pair-wise comparison (identity) of *tra* or *dsx* ORF (nucleotide) from fruit flies

Family			Tephritidae								Drosophilidae
	Genus		*Bactrocera*					*Anastrepha*		*Ceratitis*	*Drosophila*
		Species	*Bd*	*Bc*	*Bj*	*Bt*	*Bo*	*Ao*	*Af*	*Cc*	*Dm*
Tephritidae	*Bactrocera*	*Bd*		**95**	**95**	**93**	**89**	**63**	**63**	**63**	**22**
		*Bc*	98		**93**	**91**	**86**	**61**	**61**	**62**	**23**
		*Bj*	98	98		**93**	**88**	**63**	**63**	**64**	**22**
		*Bt*	97	97	98		**87**	**62**	**62**	**61**	**21**
		*Bo*	97	96	97	98		**63**	**63**	**64**	**22**
	*Anastrepha*	*Ao*	88	88	88	87	88		**98**	**64**	**22**
		*Af*	88	88	88	87	88	99		**65**	**23**
	*Ceratitis*	*Cc*	88	87	87	87	88	88	88		**21**
Drosophilidae	*Drosophila*	*Dm*	53	53	53	53	54	54	53	53	

The upper half and lower half of the table show data of *tra* (in bold) and *dsx*, respectively. The pair-wise comparisons were performed within the same genus, *Bactrocera* or *Anastrepha*, among different genera of the family Tephritidae, and with *D. melanogaster* from the family Drosophilidae fruit fly species - *Bd*, *B. dorsalis*; *Bc*, *B. correcta*; *Bj*, *B. jarvisi*; *Bt*, *B. tryoni*; *Bo*, *B. oleae*; *Ao*, *A. obliqua*; *Af*, *A. fraterculus* aff.1; *Cc*, *C. capitata*; *Dm*, *D. melanogaster*. The GenBank Accession Numbers of *tra* and *dsx* genes from studied species are consolidated in Additional file 8: Table S2 and Additional file 9: Table S3, respectively.

**Fig. 5:**
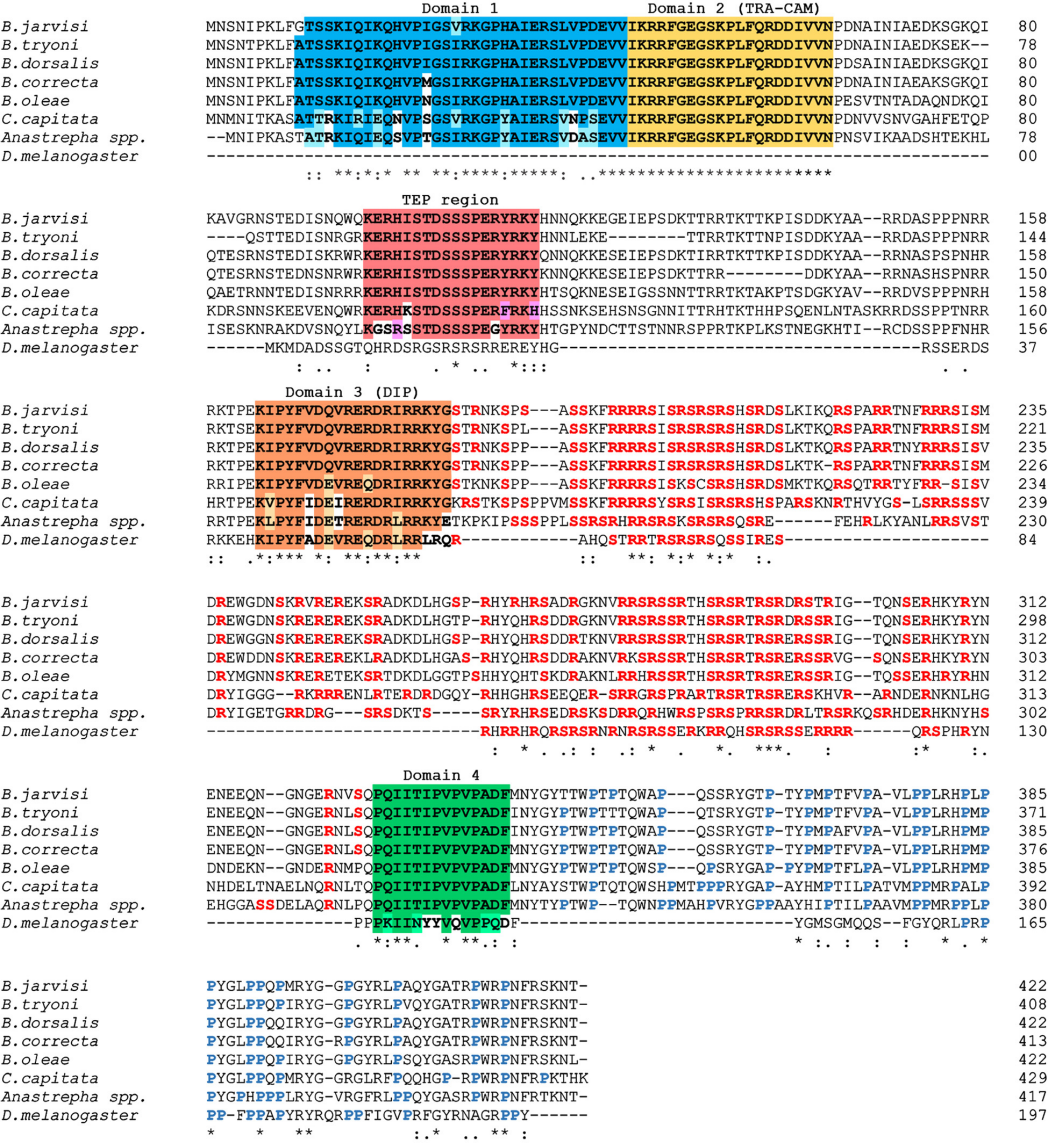
Comparison of the predicted TRA polypeptides of tephritid fruit flies and *D. melanogaster.* Fully conserved amino acid residues in domains 1, 2 (TRA-CAM), 3 (DIP), 4, and TEP region are highlighted in dense blue, yellow, brown, green, and pink, respectively while semi-conservative amino acid residues in those regions are highlighted in paler tones. Serine and arginine rich residues between domains 3 and 4 are in bold red font. The proline-rich residues after domain 4 are in bold blue. (*) and (: or .) indicate amino acids that are conserved and semi-conserved, respectively.

Five TRA polypeptides from the *Bactrocera* species, a consensus sequence from 13 *Anastrepha* species (Additional file 4: Figure S3), and *C. capitata* were aligned with *D. melanogaster* in order to identify the conserved regions (Fig. 5). The TRA protein belongs to a class of SR proteins that contains a characteristic serine-arginine dipeptide-rich region (the so-called RS domain) and a proline-rich region at the C-terminal end [11, 53]. The four known TRA specific-domains were observed (Fig. 5) [11, 54, 55]. The term “domain” used for the TRA protein studies only refers to a short region of conserved amino acid comparison [11, 54, 55]. There were no biochemical and/or functional studies of any TRA proteins. A stretch of 21 identical amino acids was confirmed as the TRA-CAM (C, *Ceratitis*; A, *Apis*; M, *Musca*) domain. The TRA-CAM domain is truncated in the male non-functional TRA protein and has been hypothesized to be involved in the autoregulation of the *tra* gene [11, 54, 55]. Domain 1 was adjacent to the TRA-CAM domain and close to the N-terminal end. It had a lesser degree of conservation (61–76 %) among tephritids. Domains 1 and 2 were absent in non-autoregulation DmTRA where the TRA protein did not act as an upstream regulator. Therefore, the TRA-CAM may play roles in receiving the primary signal from the *M* factor and/or facilitating the autoregulation as a splicing suppressor of *tra* pre-mRNA. The expected domain 3 (DIP domain) was also found to be conserved among Diptera fruit flies [11, 54, 55]. In addition, a long stretch of a serine-arginine rich region (approximately 150 amino acids) was characterized between domains 3 and 4. Twenty out of 71 (72 %) serine-arginine positions were found to be identical in the *Bactrocera* species under investigation. Only domain 4 was found to be identical in the family tephritidae. A characteristic proline-rich region (approximately 75 amino acids) at the C-terminal end was located right after domain 4. All of the conserved features were almost identical among the *Bactrocera* species.

A newly highly conserved region was discovered among tephritids with 61–83 % similarity (Fig. 5 and Additional file 5: Figure S4). This new region was named “TEP” because it appeared distinctive among tephritids. The TEP region was located approximately 33 amino acids downstream of domain 2 and was encoded by exons 2A and 2B. The TEP region may represent a specific characteristic of the TRA protein in the sex determination pathway of the tephritidae family.

The conservation level of various protein regions (i.e., domains 1 to 4, and the TEP region) within the TRA protein was as restricted as the functional protein domains found in DSX (OD1 and OD2) [44, 56] in *Bactrocera*. The term “domain” for the DSX protein has a different meaning because the OD domains are well defined protein domains supported by experimental data [57]. This fact implies that protein interactions in the sex determination pathway may be of high fidelity in function. This is especially noted for domain 2 (TRA-CAM) and domain 4 of the TRA protein, which were identical in all tephritids in this study. Although the identification of short regions showing high similarity in all tephritidae TRA proteins is interesting, these putative domains have no similarity to other proteins in metazoans. This fact diminishes the possibility to propose potential functions. In addition, the lack of any structural studies on the TRA proteins and their four putative domains is a major obstacle towards understanding these putative functional domains.

### Functional analyses of masculinization in the Salaya1 genetic sexing strain

The microinjected dsRNA containing a 1.1 kb fragment from the 3’ end of *Bdtra* cDNA can masculinize *B. dorsalis* (Additional file 6: Figure S5). In this case, the XY male- and XX female-sexual karyotypes are brown- and white-pupae, respectively, because the *B. dorsalis* Salaya 1 genetic sexing strain was used. The Salaya1 brown-pupae male contains a Y-autosome translocated chromosome which was derived from the wild-type (Phayathai1 strain) during the development of the genetic sexing strain [58]. The Y-autosome translocation chromosome was induced using a low degree of gamma radiation and underwent genetic screening that assumed the chromosomal rearrangement process. The white-pupae pseudomale can be fertile and produce daughter only progenies. However, the abnormal testes (Additional file 7: Figure S6) were also evident, as per Liu et al. 2015 [28].

**Fig. 6:**
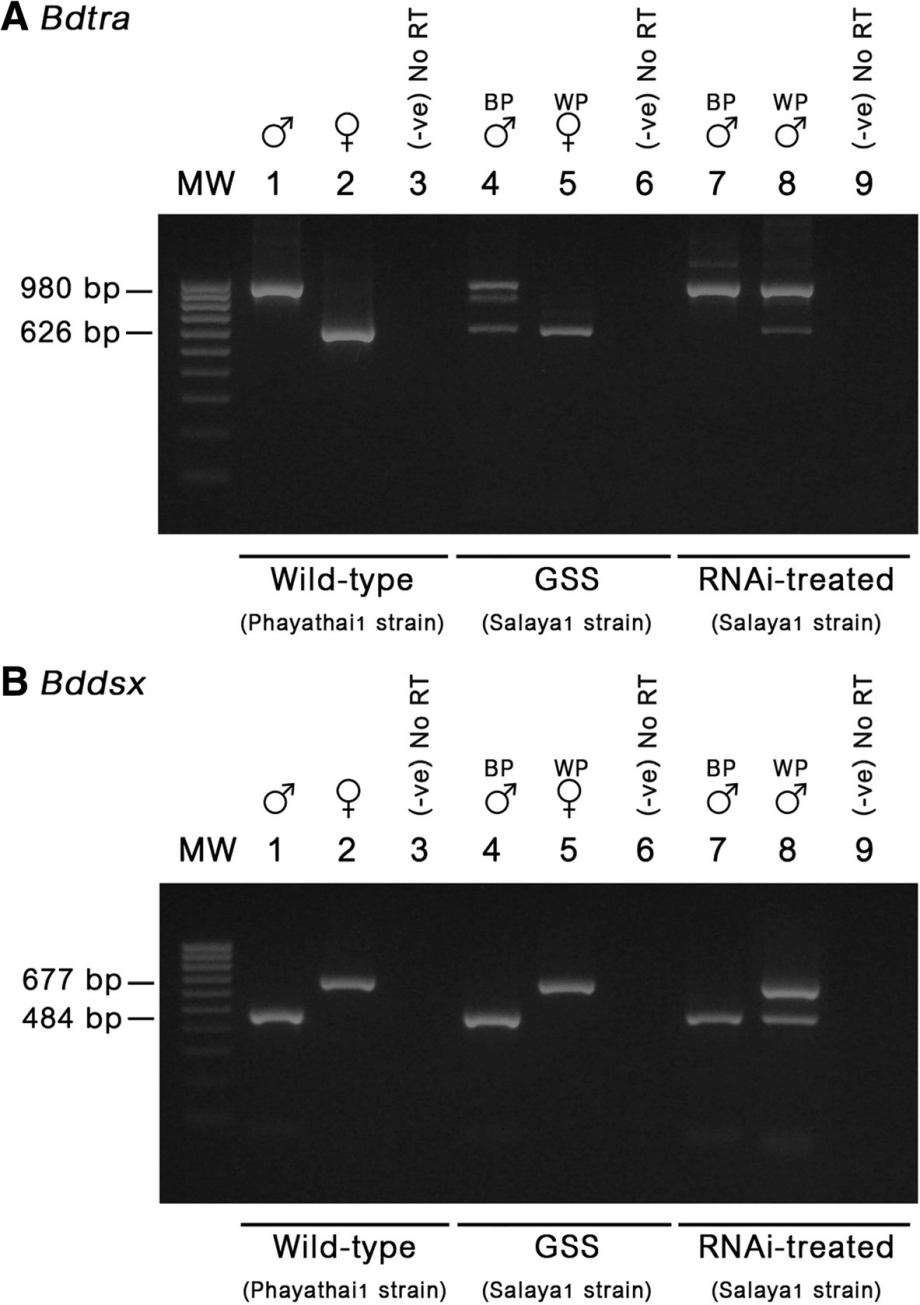
RT-PCR of sex-specific *Bdtra* and *Bddsx* transcripts in the wild strain, GSS, and *tra* RNAi-treated GSS. **a** Primers 1B-F and 2B-R1, flanking male-specific exons ms3 and ms4, were used to detect the presence of male- and female-specific *tra* transcripts (960 and 626 bp, respectively). Lanes 1, 4, and 7 are derived from male cDNA templates of wild-type, GSS (brown pupae (BP)), and RNAi-treated GSS (BP), respectively. Likewise, lanes 2 and 5 are derived from female cDNA templates of wild-type and GSS (white pupae (WP)), respectively. Lane 8 is derived from a pseudomale (WP). Lanes 3, 6, and 9 are negative controls without RT (−ve). **b** Primers *c4* (common exon) and *m* (male-specific exon) were used to detect the male-specific *dsx* transcript (484 bp). Likewise, primers *c3* (common exon) and *f* (female-specific exon) were used to detect the female-specific *dsx* transcript (677 bp) [44]. These two pairs of primers were used separately with the same cDNA templates as used in (**a**). However, the banding pattern in each lane came from the pool RT-PCR products that can detect the male- and female- specific *dsx* transcripts.

The expression of *tra* and *dsx* genes was analyzed in the male/female wild-type, the brown-pupae male/white-pupae female of the Salaya1, and *tra* RNAi- treated brown-pupae males and white-pupae pseudomales (Fig. 6). *Bdtra* primers 1B-F – 2B-R1 (this work), and *Bddsx* primers *C4* – Male-specific (*m*) primers and *C3* – Female-specific (*f*) primers [44] were used to perform the RT-PCR for individual samples. This analysis was done in order to investigate the function of the *tra* gene that regulates the sex-specific splicing of *tra* and *dsx* genes. For the *Bdtra* gene, a single band of male-specific product (980 bp) was observed in the wild-type males. Likewise, a female-specific product (626 bp) was seen in the females (Fig. 6a). All of the observed male- and female-specific products in this experiment and the following experiments were cloned for sequence confirmation. On the contrary, the coexistence of those male- and female-specific products was detected in the Salaya1 brown-pupae males. In addition, a faint intermediate band between the male- and female-specific products was consistently observed. However, this band seemed to be an artefact because only male- and female-specific products were revealed when the molecular cloning had been undertaken. Only the female-specific product was observed in the white-pupae females. In the RNAi experiment, only the male-specific product was present in the brown-pupae males after the RNAi treatment whereas the same male-specific product was detected together with a weaker signal of the female-specific product in the white-pupae pseudomales (Fig. 6a).

For the *Bddsx* gene, a single band of male-specific product (484 bp) was observed in the wild-type males and the Salaya1 brown-pupae males. Likewise, a female-specific product (677 bp) was in the females. In the RNAi experiment, only the male-specific product was also present in the brown-pupae males. However, both male- and female-specific products were detected in the white-pupae pseudomales (Fig. 6b).

For the functional analysis, RNAi experimentation confirmed that *tra* and *dsx* regulated sexual differentiation [7, 25, 26, 28, 59]. The presence of both male and female *tra* transcripts in the adult stage suggests that the sex determination and development may not be completed, considering the appearance of abnormal testis development and no mating behavior in most of the pseudomales.

The hypothetical Y-linked male determining factor (*M* factor) is proposed as that which initiates the blocking of *tra* autoregulatory loops and prohibits female-specific splicing of the zygotic *tra* pre-mRNA in precellular blastoderm male embryos of *C. capitata* and *Bactrocera jarvisi* (Tryon) [6, 8]. During this period, the translation of the inactive TRA protein increases and abolishes the *tra* autoregulatory loop in males because there is no functional splicing regulation from TRA/TRA-2 complex binding. The abundance of zygotic male-specific, female-specific, and heterogeneous *tra* mRNA is apparent in both male and female embryos during this primary signalling [6, 28]. Afterward, the male-specific and female-specific *tra* transcripts are generally and exclusively established in males and females, respectively, presumably because of the stable genetic switch *tra*.

The coexistence of male- and female-specific *tra* transcripts during the same period could not be confirmed in the expression analysis of precellularized *B. dorsalis* embryos because the embryos were not sex sorted [28]. However, the presence of the male-specific transcript similarly appears before cellularization. This suggests that the roles of the non-characterized, but postulated, *M* factor may be highly conserved in *Bactrocera* and tephritid fruit flies [6–8, 25–28]. The introgression of the presumably *M* factor-bearing Y-chromosome from *B. jarvisi* and *B. dorsalis* into *B. tryoni* and *B. carambolae*, respectively, could interchangeably regulate normal sex determination [8, 60]. However, in this work, the mixed male- and female-specific *tra* transcripts were observed in all of the brown-pupae adult (XY) males but not in the white-pupae (XX) females of the Salaya1 strain at the adult stage. We hypothesized that the *M* factor on the Y-chromosome had acquired a leaky mutant stage from the Y-chromosome rearrangement originating from the GSS construction; the *M* factor cannot completely block the autoregulatory loop in all male karyotype individuals. The leaky *M* factor function can be rescued by *tra* RNAi-treatment in the brown-pupae adult males. This results in the disappearance of female-specific *tra* transcripts. The situation can also be partly imitated in the application of *tra* RNAi to the white pupae (XX pseudomales). However, a clear difference is that the same coexistence of both male- and female-specifically spliced *tra* transcripts did not result in the same co-expression of both male- and female-specifically spliced *dsx* transcripts as in the case of the Salaya1 male (with the *M* factor) versus the pseudomale (without the *M* factor). In the pseudomale, the incomplete expression of a functional TRA protein can form a TRA/TRA-2 complex which is effective enough to control the female-specifically spliced *dsx*. However, in the Salaya1 male that has a leaky *M* factor, the similarly incomplete expression of a functional TRA protein may also form a TRA/TRA-2 complex, but it seems that the TRA/TRA-2 complex is not effective enough to activate a female-specifically spliced *dsx.* This fact suggests a possible direct association between the presences of the *M* factor and TRA-2 gene in the adult stage. In order to provide a justification, we postulate that the *M* factor may extend its function into adult males by inactivation of not only TRA, but also TRA-2 proteins. A more recent male sex determination model proposed by Salvemini et al. 2009 [25] suggests the possibility of an *M* factor repressed TRA-2 protein/gene at the embryonic stage. We propose that this mechanism can function during the adult stage. It is proposed that the *M* factor directly inhibits TRA protein activation in *C. capitata* [6, 7] and *Bactrocera* species [8]. If the leaky mutant *M* factor in the Salaya1 brown-pupae males affects the TRA and TRA-2 protein activities in adult males, the level of TRA/TRA-2 complexes may not reach the threshold to enhance female-specific exonic splicing of *dsx* pre-mRNA.

### Potential application of *tra* genes for the development of a genetic sexing system in SIT programs

The comparative study of *tra* in many *Bactrocera* species suggests a practical application of the *tra* based-genetic tools. The cross-species utilization of a very highly conserved male-specific exon/intron cassette in the generation of a sex-specific lethality strain is likely among the *Bactrocera* genera. The TRA/TRA-2 binding sites located in the last male-specific exon of *tra* genes are identical in *B. dorsalis* (this work), *B. correcta* (this work), *B. zonata* [61], *B. tryoni* [8], *B. jarvisi* [8], and *B. oleae* [26]. Therefore, a single construct for female-specific RIDL (Release of Insects carrying a Dominant Lethal) or fsRIDL may work very well for various *Bactrocera* pests because it relies on the male-specific exon splicing of the *tra* gene [62, 63]. The *tra* RNAi-treatment can be generally used for sexual transformation. However, the derived pseudomales may not be fully masculinized with normal reproductive potential as in this work and elsewhere [7, 25, 26, 28, 59]. Further studies of the sex determination pathway, especially the finding and characterization of the male-determining loci on Y-chromosome and its primary signal pathways would lead to an ideal tool to develop a new masculinization strategy for the genetic sexing strain. The putative core promoters and their upstream regions should contain all necessary promoter/enhancer *cis*-regulatory elements because these regions are all flanked by the highly conserved LOC 105232903 orthologues. The potential application of the promoter is for it to be used in an early embryonic driver constructions, such as a tetracycline (Tet)-suppressible system [62, 64–66], because the zygotic *tra* expression is a very early event, before the cellularization stage.

## Conclusions

The comparison of *Bdtra* and *Bctra* genes to the other *Bactrocera* and tephritid flies confirms the conserved functional roles of *tra* in the sex determination pathway. In addition, a new TEP region is proposed because of the conservation among tephritid fruit flies. The generation of new male-specific splicing exons in the *Bdtra* supports that the initial stage of the alternative splicing process involves multiple exon retention. The evolution of sex-specific splicing is radiating within species from the *B. dorsalis* model. All of the 5’ regulatory regions of the *Bactrocera tra* genes contain characteristics of the putative core promoter. The comparative molecular features of *Bdtra* and *Bctra* also offer potential applications directly for the *B. dorsalis*, *B. correta*, or universally for other *Bactrocera* genetic sexing systems for SIT.

## Methods

### Fruit fly strains and specimens

The laboratory stocks, *B. dorsalis* (Phayathai1 strain) and *B. correcta* (Phayathai2 strain), were used to isolate and characterize *Bdtra* and *Bctra* genes, respectively. The *B. dorsalis* Salaya1 GSS is based on the brown-white pupal color dimorphisms (i.e., males and females that emerged from brown and white pupae, respectively [58]). They were used for expression and functional analyses of the *Bdtra* gene. The fruit flies were reared at 25 °C and 13 L: 11D cycles. *B. zonata* and *B. tryoni* specimens were from laboratory stocks of the Insect Pest Control Laboratory of the International Atomic Energy Agency, Seiberdorf, Austria. In addition, samples of *B. carambolae* were from the Jakarta strain.

### GenBank Accession Numbers

The GenBank Accession Numbers of *tra* and *dsx* genes used in this study were from various tephritid species and *D. melanogaster.* The *tra* and *dsx* orthologues were separately grouped and consolidated in Additional file 8: Table S2 and Additional file 9: Table S3, respectively.

### DNA and RNA extractions

Genomic DNA was individually extracted from adult fruit flies according to Baruffi et al. 1995 [67]. Total RNA was isolated from adult male and female fruit flies (2–4 days old) using TRIzol® reagent (Invitrogen, USA) as per the manufacturer’s instructions.

### Isolation of *Bdtra* and *Bctra*

The primers BoF and BoR designed from *Botra* [GenBank: AJ715413], were used to firstly isolate the *tra* orthologues of *B. dorsalis* and *B. correcta*, using genomic DNA as templates. All primers used in this work are described in Additional file 10: Table S4. The partial sequences from both species were aligned to derive primers for 3’RACE and nested PCR. Three to five micrograms of total RNA were reverse transcribed to first strand cDNA using the oligo (dT) adapter primer and ImProm-II™ Reverse Transcriptase (Promega, USA), following the manufacturer’s instructions. One-tenth of the initial reverse transcription reaction was used as a template in 3’ RACE using 2B-F and adapter primers. Amplification was performed using a FlexCycler PCR thermal cycler (Analytik Jena, Germany) with the following conditions: 94 °C for 4 min, held at 72 °C for 5 min while *Taq* polymerase (Vivantis, Malaysia) was added, then one cycle of 60 °C for 2 min and 72 °C for 2 min, followed by five cycles of 94 °C for 1 min, 60 °C for 2 min, 72 °C for 2 min; 28 cycles of 91 °C for 40 s, 58 °C for 2 min, 72 °C for 2 min; and one cycle of final extension at 72 °C for 7 min. The negative controls for each set of RT-PCR experiments were performed excluding reverse transcriptase in order to ensure no genomic DNA contamination.

For the characterization of the 5’ UTR regions and the translational start site of *Bdtra*, two sets of RT-PCR [(1B-F – 2B-R1) and (5U-BD1 – 2A-2R)] were performed to generate two contiguous fragments to assemble the 5’UTR region and translational start site. Cycling conditions were as follows: 94 °C for 2 min; 29 cycles of 94 °C for 1 min, 60 °C for 30 s, 72 °C for 2 min; and one cycle of final extension at 72 °C for 7 min. PCR was also performed on genomic DNA templates to determine the exon/intron junctions. Three sets of primers (i.e., 5U-BD1 – 2A-2R, 1B-TraF – 2B-R2, and Nested TraF – 3Tra-R) were used to amplify the contiguous fragments with the following cycling conditions: 94 °C for 5 min; 29 cycles of 94 °C for 1 min, 60 °C for 30 s, 72 °C for 1 min; and one cycle of final extension at 72 °C for 7 min. For the same *Bctra* characterization, the previously described procedures were carried out, except that the primer 5U-BD1 was replaced by 5U-BC1.

### Inverse PCR

*Bdtra* and *Bctra* sequences (this work) were inspected to identify the proper restriction enzymes. Subsequently, primers were designed based on the 5’UTR regions. The inverse PCR method was as per Permpoon et al. 2011 [44] except *Eco*RI restriction digest. Inverse PCR was carried out with 5U-BD1_R – 5U-BD3 primers (for *B. dorsalis*) and 5U-BC1_R – 5U-BC4 primers (for *B. correcta*) using the following cycling conditions: 94 °C for 7 min; 29 cycles of 94 °C for 1 min, 58 °C for 30 s, 72 °C for 5 min; and one cycle of final extension at 72 °C for 10 min.

### Functional promoter analysis

A 1.4 kb fragment of the 5’ upstream regulatory region including the putative core promoter and translational start codon of *Bctra* gene was amplified with the BcT-3_*Nco*I – BcT-4_*Xba*I primers using genomic DNA as a template. Cycling conditions were as follows: 94 °C for 5 min; 29 cycles of 94 °C for 1 min, 58 °C for 30 s, 72 °C for 2 min; and one cycle of final extension at 72 °C for 7 min. The amplicon was subsequently cloned into the pGEM®-T Easy Vector (Promega, USA). The *Nco*I-*Xba*I cut *Bctra* fragments were ligated in the *Nco*I-*Xba*I cut #1215 vector (pSL*af tTA-SV40_af*) [65] to create the #1215-*Bctra* vector (pSL*af_Bctra–tTA_af*). A *DsRed* restriction fragment was generated from dsRed_*Xba*IF – dsRed_*BamH*IR primers using the #1200 vector (p*Bac*[*fa PUb-DsRed*]) as a template [68]. The *Bctra*-*DsRed* construct was created by molecular cloning replacement of the *Xba*I-*BamH*I tTA fragment with the *Xba*I-*BamH*I DsRed fragment into the #1215-*Bctra* vector and called #1215-*Bctra-DsRed* vector (pSL*af_Bctra–DsRed_af*).

The transient expression was performed by microinjection of the #1215-*Bctra*-*DsRed* constructs (750 ng/µl) into the dechorionated precellular blastoderm embryos from the *B. correcta* wild-type strain. The microinjected embryos representing developmental times (from 1 to 48 hours after egg laying (h AEL)) were screened every hour by epifluorescence for the expression of the #1215-*Bctra*-*DsRed* construct and compared with the uninjected embryos. The screening was performed using the fluorescence stereomicroscope Olympus SZX16 Reflected Fluorescence System with the filters SZX2-FRFP (BP545-580; BA610IF). Photographic documentation was done using an Olympus DP2-BSW version 2.2.

### Isolation of additional 5’ upstream putative regulatory region of *tra* genes from *B. zonata*, *B. tryoni*, and *B. carambolae*

The forward primer Tpro was designed based on putative coding sequences of the LOC105232903 locus [GenBank: XM_011214797.1] from *B. dorsalis* because the sequence was found to be immediately upstream of the 5’ regulatory region of the *Bdtra* and *Bctra* in this work. The reverse primer 1B-1R was designed on the basis of the conserved *tra* coding sequences approximately 150 bp from the start codon. The genomic DNA of *B. zonata*, *B. tryoni*, and *B. carambolae* were used as templates. The PCR conditions were as per the following: 94 °C for 5 min; 29 cycles of 94 °C for 1 min, 58 °C for 30 s, 72 °C for 2 min; and one cycle of final extension at 72 °C for 7 min. The 5’ and 3’ sequences of the derived specific PCR products were validated for being the corresponding CDS of the LOC105232903 locus and *tra* orthologues by multiple sequence alignment with the homologous regions in *B. dorsalis* and *B. correcta*.

### Cloning PCR fragments

The specific amplicon was purified with the QIAquick gel cleanup kit (QIAGEN, Germany). The products were cloned into the pGEM®-T Easy vector (Promega, USA) according to the manufacturer’s instructions. The recombinant plasmids were transformed into DH5α competent cells and screened. Plasmids were extracted as described by Sambrook et al. 1989 [69]. All sequencing reactions were performed on both strands using the sequencing service from Macrogen Inc., Seoul, Korea.

### RT-PCR expression analysis

The first stand cDNA of both sexes from each species was separately synthesized. The reverse transcription method and the negative controls were similar to the *Bdtra* and *Bctra* isolation procedure. The RT-PCR expression analysis was performed using common and sex-specific primers, represented in Fig. 2a. Cycling conditions were as follows: 94 °C for 2 min; 29 cycles of 94 °C for 1 min, 60 °C for 30 s, 72 °C for 1 min; and one cycle of final extension at 72 °C for 7 min. All of the RT-PCR experiments were repeated three times. The primers used in this study are described in Additional file 10: Table S4.

### RNAi experiment

The 2B-F and adapter primers located on exon 2B and the 3’ end poly (A) tail, respectively, were used to amplify an approximately 1.1 kb fragment of the *Bdtra*. The amplicon (forward gene fragment) and the inverted sequence (reverse gene fragment) were cloned into pGEM®-T easy vector (Promega, USA) and transformed into *E. coli* (DH5α). Single-stranded RNA (ssRNA) of *Bdtra* was synthesized by *in vitro* bacterial expression using the T7-polymerase system. To produce double-stranded RNA (dsRNA), forward and reverse RNA fragments were mixed in annealing buffer (10 mM Tris pH 8 and 10 mM NaCl). Incubation was done at 75 °C for 5 min (annealing), then cooled down to room temperature (25 °C).

The Salaya1 embryos were harvested and kept for 40 min at 18 °C. Approximately 1000 precellular blastoderm embryos were dechorionated and micro-injected with 1 μg/μl of *Bdtra* dsRNA at the posterior end. The surviving RNAi-treated embryos were raised to the pupal stage in normal conditions. The brown and white pupae were individually isolated, awaiting sexual identification after adult emergence. The white-pupae males were categorized as pseudomales because the untreated RNAi white pupae were females with the XX sexual karyotype [58].

Mating behavior and fertility of the sexually mature pseudomales were tested against RNAi-treated and RNAi-untreated brown-pupae males from the Salaya1 strain. The investigation was carried out by pairing individual tested males or pseudomales with three normal sexually mature and virgin white-pupae females. Five RNAi-untreated brown-pupae males, five RNAi-treated brown-pupae males, and six RNAi-treated white-pupae pseudomales were individually tested for mating behavior and fertility when they were sexually mature. Subsequently, their testis development was inspected by microscopic dissection.

### RT-PCR analysis of RNAi-treated flies

The previously described RT-PCR conditions were performed to analyze the sex-specific expression patterns of *Bdtra* and *Bddsx* from *B. dorsalis* (Phayathai1 and Salaya1 strains) and their RNAi-treated brown-pupae males versus white-pupae pseudomales. Three sets of primers were used in this experiment: 1B-F – 2B-R1 primers for *Bdtra* analysis, *C3* – Female-specific (*f*) primers for female-specific *Bddsx* analysis, and *C4* – Male-specific (*m*) primers for male-specific *Bddsx* analysis [44]. All of the RT-PCR experiments were repeated three times. The primer sequences are presented in Additional file 10: Table S4.

### Sequence alignment and phylogenetic tree reconstruction

Sequences were searched using the BLAST family of programs from the NCBI database for gene identification. Genomic DNA and cDNA sequences were aligned using the ClustalX2 program [70] and Unipro UGENE Version 1.14.0 [71]. The genetic distance was estimated using Jukes-Cantor measurement. The phylogenetic tree of *tra* genes were reconstructed using the UPGMA method in the CLC Main Workbench version 7.6.2 (CLC Bio, QIAGEN). The bootstrap values were formulated from 1000 replicates with the 50 % majority rule.

## Ethics statement

This manuscript describes functional genetics of non-regulated invertebrate (domestic insect pests) thus did not require animal ethics approval.

## Availability of data

All supporting data are included as additional files.

## Additional files

Additional file 1: Figure S1. Comparison of putative *cis*-regulatory elements for sex-specific splicing of *tra* genes in *Bactrocera* species. (A) Localization and number of putative TRA/TRA-2 binding sites (green ovals), RBP1 binding sites (blue ovals), TRA-2 ISS sequences (red rectangles), and purine-rich elements (brown triangles) are indicated in the male-specific exons and the respective flanking introns of *B. dorsalis* (this work), *B. correcta* (this work), *B. zonata* [61], *B. tryoni* [8], *B. jarvisi* [8], and *B. oleae* [26]. The number below each element corresponds to sequences in Additional file 2: Table S1. (B) The consensus sequence of each *cis*-regulatory element grouping is based on the following sources: *D. melanogaster* [32], *Anastrepha* species [27], *C. capitata* [7], and *Bactrocera* species. (PDF 248 kb) Additional file 2: Table S1. Sequence of the putative TRA/TRA-2 binding sites, RBP1 binding sites, TRA-2 ISS sequences, and purine-rich elements. (PDF 219 kb) Additional file 3: Figure S2. Molecular phylogeny reconstructed from the female *tra* CDS. The tree was plotted using the UPGMA method. The horizontal branch-lengths are proportional to the genetic distance based on Jukes-Cantor measurement. The numbers shown at branch points correspond to bootstrap values from 1000 replicates with the 50 % majority rule. (PDF 821 kb) Additional file 4: Figure S3. Aligned sequences of putative TRA polypeptides of the *Anastrepha* species. The consensus sequences of *Anastrepha* species are represented in bold text above each line, and the points stand for the same amino acids. The GenBank Accession Numbers of *Anastrepha tra* genes were consolidated in Additional file 8: Table S2. (PDF 458 kb) Additional file 5|: Figure S4. TEP region of TRA protein alignment from tephritid fruit flies. The TEP region is in bold text and highlighted in pink. The identical amino acids (*) are in heavy shade. The semi-conservative amino acids (: or .) are highlighted in medium shade while the less conserved regions are in the lightest shade. (PDF 4253 kb) Additional file 6: Figure S5. Phenotypic analysis of male genitalia from *tra* RNAi-treated pseudomales. (A) and (B) represent the ventral view of male and female individuals, respectively, from the wild-type (wt) *B. dorsalis* (Phayathai1 strain) while (C) and (D) illustrate a closer look at the normal male and female genitalia, respectively. (E) and (F) are also respectively the genitalia and ovipositor from a brown-pupae male and white-pupae female of the Salaya1 (SY1), GSS. (G) is the genitalia of the *tra* RNAi-treated brown-pupae male whereas (H) to (J) are the genitalia of the *tra* RNAi-treated white-pupae pseudomale. (XX) and (XY) are presumably sexual karyotypes of each individual sub-figure. (JPG 1087 kb) Additional file 7: Figure S6. The development of testes dissected from males and pseudomales. (A), (B), (C), and (D) represent a pair of fertile and fully developed testes from a wild-type (wt) male (Phayathai1 strain), a brown-pupae male (Salaya1 (SY1), GSS), a *tra* RNAi-treated brown-pupae male, and a *tra* RNAi-treated white-pupae pseudomale, respectively. (E) to (H) show a pair of sterile and aberrant testes from *tra* RNAi-treated white-pupae pseudomales that had no observed courtship behavior. (XX) and (XY) are presumably sexual karyotypes of each individual sub-figure. (JPG 659 kb) Additional file 8: Table S2. GenBank Accession Numbers of *transformer* genes in Tephritid species and *Drosophila melanogaster*. (PDF 94 kb) Additional file 9: Table S3. GenBank Accession Numbers of *doublesex* genes in Tephritid species and *Drosophila melanogaster*. (PDF 87 kb) Additional file 10: Table S4. Sequence of primers used in this study. (PDF 78 kb)

## References

[1] Darwin C (1977). Principles of sexual selection. The origin of species and the descent of man.

[2] Bachtrog D, Mank JE, Peichel CL, Kirkpatrick M, Otto SP, Ashman T-L (2014). Sex determination: Why so many ways of doing it?. PLoS Biol.

[3] Sánchez L (2008). Sex-determining mechanisms in insects. Int J Dev Biol.

[4] Bopp D, Saccone G, Beye M (2014). Sex determination in insects: variations on a common theme. Sex Dev.

[5] Cline TW (1978). Two closely linked mutations in *Drosophila melanogaster* that are lethal to opposite sexes and interact with daughterless. Genetics.

[6] Gabrieli P, Falaguerra A, Siciliano P, Gomulski L, Scolari F, Zacharopoulou A (2010). Sex and the single embryo: early development in the Mediterranean fruit fly, *Ceratitis capitata*. BMC Dev Biol.

[7] Pane A, Salvemini M, Bovi PD, Polito C, Saccone G (2002). The *transformer* gene in *Ceratitis capitata* provides a genetic basis for selecting and remembering the sexual fate. Development.

[8] Morrow JL, Riegler M, Frommer M, Shearman DCA (2014). Expression patterns of sex-determination genes in single male and female embryos of two *Bactrocera* fruit fly species during early development. Insect Mol Biol.

[9] Morrow JL, Riegler M, Gilchrist AS, Shearman DCA, Frommer M (2014). Comprehensive transcriptome analysis of early male and female *Bactrocera jarvisi* embryos. BMC Genet.

[10] Hilfiker-Kleiner D, Dubendorfer A, Hilfiker A, Nothiger R (1993). Developmental analysis of two sex-determining genes, *M* and *F*, in the housefly, *Musca domestica*. Genetics.

[11] Hediger M, Henggeler C, Meier N, Perez R, Saccone G, Bopp D (2010). Molecular characterization of the key switch *F* provides a basis for understanding the rapid divergence of the sex-determining pathway in the housefly. Genetics.

[12] Shukla JN, Palli SR (2012). Sex determination in beetles: production of all male progeny by parental RNAi knockdown of *transformer*. Scientific Reports.

[13] Verhulst EC, Lynch JA, Bopp D, Beukeboom LW, van de Zande L (2013). A new component of the *Nasonia* sex determining cascade is maternally silenced and regulates *transformer* expression. PLoS ONE.

[14] Gempe T, Hasselmann M, Schiøtt M, Hause G, Otte M, Beye M (2009). Sex determination in honeybees: two separate mechanisms induce and maintain the female pathway. PLoS Biol.

[15] Kiuchi T, Koga H, Kawamoto M, Shoji K, Sakai H, Arai Y (2014). A single female-specific piRNA is the primary determiner of sex in the silkworm. Nature.

[16] Erickson JW, Quintero JJ (2007). Indirect effects of ploidy suggest X chromosome dose, not the X:A ratio, signals sex in *Drosophila*. PLoS Biol.

[17] Salz HK, Erickson JW (2010). Sex determination in *Drosophila*: the view from the top. Fly.

[18] Schutt C, Nothiger R (2000). Structure, function and evolution of sex-determining systems in Dipteran insects. Development.

[19] Keyes LN, Cline TW, Schedl P (1992). The primary sex determination signal of *Drosophila* acts at the level of transcription. Cell.

[20] Bell LR, Horabin JI, Schedl P, Cline TW (1991). Positive autoregulation of *Sex-lethal* by alternative splicing maintains the female determined state in *Drosophila*. Cell.

[21] Inoue K, Hoshijima K, Sakamoto H, Shimura Y (1990). Binding of the *Drosophila Sex-lethal* gene product to the alternative splice site of *transformer* primary transcript. Nature.

[22] Valcárcel J, Singh R, Zamore PD, Green MR (1993). The protein Sex-lethal antagonizes the splicing factor U2AF to regulate alternative splicing of *transformer* pre-mRNA. Nature.

[23] Bopp D, Bell LR, Cline TW, Schedl P (1991). Developmental distribution of female-specific Sex-lethal proteins in *Drosophila melanogaster*. Genes Dev.

[24] Willhoeft U, Franz G (1996). Identification of the sex-determining region of the *Ceratitis Capitata Y* chromosome by deletion mapping. Genetics.

[25] Salvemini M, Robertson M, Aronson B, Atkinson P, Polito LC, Saccone G (2009). *Ceratitis capitata transformer-2* gene is required to establish and maintain the autoregulation of *Cctra*, the master gene for female sex determination. Int J Dev Biol.

[26] Lagos D, Koukidou M, Savakis C, Komitopoulou K (2007). The *transformer* gene in *Bactrocera oleae*: the genetic switch that determines its sex fate. Insect Mol Biol.

[27] Ruiz MF, Milano A, Salvemini M, Eirín-López JM, Perondini ALP, Selivon D (2007). The gene *transformer* of *Anastrepha* fruit flies (Diptera, Tephritidae) and its evolution in insects. PLoS ONE.

[28] Liu G, Wu Q, Li J, Zhang G, Wan F (2015). RNAi-mediated knock-down of *transformer* and *transformer 2* to generate male-only progeny in the oriental fruit fly, *Bactrocera dorsalis* (Hendel). PLoS ONE.

[29] Peng W, Zheng W, Handler A, Zhang H (2015). The role of the transformer gene in sex determination and reproduction in the tephritid fruit fly, *Bactrocera dorsalis* (Hendel). Genetica.

[30] Sarno F, Ruiz MF, Eirín-López JM, Perondini ALP, Selivon D, Sánchez L (2010). The gene *transformer-2* of *Anastrepha* fruit flies (Diptera, Tephritidae) and its evolution in insects. BMC Evol Biol.

[31] Saccone G, Salvemini M, Polito LC (2011). The *transformer* gene of *Ceratitis capitata*: a paradigm for a conserved epigenetic master regulator of sex determination in insects. Genetica.

[32] Burtis KC, Baker BS (1989). *Drosophila doublesex* gene controls somatic sexual differentiation by producing alternatively spliced mRNAs encoding related sex-specific polypeptides. Cell.

[33] Hoshijima K, Inoue K, Higuchi I, Sakamoto H, Shimura Y (1991). Control of *doublesex* alternative splicing by *transformer* and *transformer-2* in *Drosophila*. Science.

[34] Shukla JN, Nagaraju J (2010). *Doublesex*: a conserved downstream gene controlled by diverse upstream regulators. J Genet.

[35] Hedley ML, Maniatis T (1991). Sex-specific splicing and polyadenylation of *dsx* pre-mRNA requires a sequence that binds specifically to *tra-2* protein in vitro. Cell.

[36] Tian M, Maniatis T (1993). A splicing enhancer complex controls alternative splicing of *doublesex* pre-mRNA. Cell.

[37] Saccone G, Peluso I, Testa G, Di Paola F, Pane A, Polito LC. *Drosophila Sex-lethal* and *doublesex* homologous genes in *Ceratitis capitata*: searching for sex-specific genes to develop a medfly transgenic sexing strain, in enhancement of the sterile insect technique through genetic transformation using nuclear techniques. Vienna: IAEA/FAO; 1996.

[38] Saccone G, Pane A, Testa G, Santoro M, De Martino G, Di Paola F, Tan KH (2000). Sex determination in medfly: a molecular approach. Area-wide control of fruit flies and other insect pests.

[39] Shearman DCA, Frommer M (1998). The *Bactrocera tryoni* homologue of the *Drosophila melanogaster* sex-determination gene *doublesex*. Insect Mol Biol.

[40] Lagos D, Ruiz MF, Sánchez L, Komitopoulou K (2005). Isolation and characterization of the *Bactrocera oleae* genes orthologous to the sex determining *Sex-lethal* and *doublesex* genes of *Drosophila melanogaster*. Gene.

[41] Ruiz MF, Stefani RN, Mascarenhas RO, Perondini ALP, Selivon D, Sánchez L (2005). The gene *doublesex* of the fruit fly *Anastrepha obliqua* (Diptera, Tephritidae). Genetics.

[42] Ruiz M, Eirín-López J, Stefani R, Perondini AP, Selivon D, Sánchez L (2007). The gene *doublesex* of *Anastrepha* fruit flies (Diptera, Tephritidae) and its evolution in insects. Dev Genes Evol.

[43] Chen S-L, Dai S-M, Lu K-H, Chang C (2008). Female-specific *doublesex* dsRNA interrupts yolk protein gene expression and reproductive ability in oriental fruit fly, *Bactrocera dorsalis* (Hendel). Insect Biochem Mol Biol.

[44] Permpoon R, Aketarawong N, Thanaphum S (2011). Isolation and characterization of *Doublesex* homologues in the *Bactrocera* species: *B. dorsalis* (Hendel) and *B. correcta* (Bezzi) and their putative promoter regulatory regions. Genetica.

[45] White IM, Elson-Harris MM (1992). Fruit flies of economic significance: their identification and bionomics.

[46] Ast G (2004). How did alternative splicing evolve?. Nat Rev Genet.

[47] Qi J, Su S, Mattox W (2007). The *doublesex* splicing enhancer components Tra2 and Rbp1 also repress splicing through an intronic silencer. Mol Cell Biol.

[48] Heinrichs V, Baker BS (1995). The *Drosophila* SR protein RBP1 contributes to the regulation of *doublesex* alternative splicing by recognizing RBP1 RNA target sequences. EMBO J.

[49] Heinrichs V, Ryner LC, Baker BS (1998). Regulation of Sex-Specific Selection of *fruitless* 5′ Splice Sites by *transformer* and *transformer*-2. Mol Cell Biol.

[50] Juven-Gershon T, Kadonaga JT (2010). Regulation of gene expression via the core promoter and the basal transcriptional machinery. Dev Biol.

[51] Butler JEF, Kadonaga JT (2002). The RNA polymerase II core promoter: a key component in the regulation of gene expression. Genes Dev.

[52] Kutach AK, Kadonaga JT (2000). The downstream promoter element DPE appears to be as widely used as the TATA box in *Drosophila* core promoters. Mol Cell Biol.

[53] Manley JL, Tacke R (1996). SR proteins and splicing control. Genes Dev.

[54] Verhulst EC, van de Zande L, Beukeboom LW (2010). Insect sex determination: it all evolves around *transformer*. Curr Opin Genet Dev.

[55] Geuverink E, Beukeboom LW (2014). Phylogenetic distribution and evolutionary dynamics of the sex determination genes *doublesex* and *transformer* in insects. Sex Dev.

[56] Permpoon R, Thanaphum S (2010). Isolation and characterization of oligomerization domain I and II coding regions of *doublesex* genes in agricultural fruit flies (Diptera: Tephritidae). Eur J Entomol.

[57] Yang Y, Zhang W, Bayrer JR, Weiss MA (2008). *Doublesex* and the regulation of sexual dimorphism in *Drosophila melanogaster*: structure, function, and mutagenesis of a female-specific domain. J Biol Chem.

[58] Isasawin S, Aketarawong N, Thanaphum S (2012). Characterization and evaluation of microsatellite markers in a strain of the oriental fruit fly, *Bactrocera dorsalis* (Diptera: Tephritidae), with a genetic sexing character used in sterile insect population control. Eur J Entomol.

[59] Schetelig MF, Milano A, Saccone G, Handler AM (2012). Male only progeny in *Anastrepha suspensa* by RNAi-induced sex reversion of chromosomal females. Insect Biochem Mol Biol.

[60] Isasawin S, Aketarawong N, Lertsiri S, Thanaphum S (2014). Development of a genetic sexing strain in *Bactrocera carambolae* (Diptera: Tephritidae) by introgression of sex sorting components from *B. dorsalis*, Salaya1 strain. BMC Genet.

[61] Leftwich PT, Koukidou M, Rempoulakis P, Gong H-F, Zacharopoulou A, Fu G, et al. Genetic elimination of field-cage populations of Mediterranean fruit flies. Proc Roy Soc Lond B Biol Sci. 2014. 281(1792). 10.1098/rspb.2014.1372 PMC4150327 25122230

[62] Koukidou M, Alphey L (2014). Practical applications of insects’ sexual development for pest control. Sex Dev.

[63] Ant T, Koukidou M, Rempoulakis P, Gong H-F, Economopoulos A, Vontas J (2012). Control of the olive fruit fly using genetics-enhanced sterile insect technique. BMC Biol.

[64] Dafa’alla T, Fu G, Alphey L (2010). Use of a regulatory mechanism of sex determination in pest insect control. J Genet.

[65] Schetelig MF, Caceres C, Zacharopoulou A, Franz G, Wimmer EA (2009). Conditional embryonic lethality to improve the sterile insect technique in *Ceratitis capitata* (Diptera: Tephritidae). BMC Biol.

[66] Schetelig MF, Handler AM (2012). Strategy for enhanced transgenic strain development for embryonic conditional lethality in *Anastrepha suspensa*. Proc Natl Acad Sci U S A.

[67] Baruffi L, Damiani G, Guglielmino CR, Bandi C, Malacrida AR, Gasperi G (1995). Polymorphism within and between populations of *Ceratitis capitata*: comparison between RAPD and multilocus enzyme electrophoresis data. Heredity.

[68] Scolari F, Schetelig MF, Bertin S, Malacrida AR, Gasperi G, Wimmer EA (2008). Fluorescent sperm marking to improve the fight against the pest insect *Ceratitis capitata* (Wiedemann; Diptera: Tephritidae). New Biotechnol.

[69] Sambrook J, Fitsch EF, Maniatis T (1989). Molecular Cloning: A Laboratory Manual.

[70] Larkin MA, Blackshields G, Brown NP, Chenna R, McGettigan PA, McWilliam H (2007). Clustal W and Clustal X version 2.0.. Bioinformatics.

[71] Okonechnikov K, Golosova O, Fursov M, UTENE team (2012). Unipro UGENE: a unified bioinformatics toolkit. Bioinformatics.

