# Supplementary material for: The study of the transformer gene from Bactrocera dorsalis and B. correcta with putative core promoter regions
Source: BMC Genet. 2016 Feb 1;17:34. doi: 10.1186/s12863-016-0342-0 (PMC4736151; doi:10.1186/s12863-016-0342-0)
Supplement: Additional file 1: Figure S1. — Comparison of putative cis-regulatory elements for sex-specific splicing of tra genes in Bactrocera species. (A) Localization and number of putative TRA/TRA-2 binding sites (green ovals), RBP1 binding sites (blue ovals), TRA-2 ISS sequences (red rectangles), and purine-rich elements (brown triangles) are indicated in the male-specific exons and the respective flanking introns of B. dorsalis (this work), B. correcta (this work), B. zonata [61], B. tryoni [8], B. jarvisi [8], and B. oleae [26]. The number below each element corresponds to sequences in Additional file 2: Table S1. (B) The consensus sequence of each cis-regulatory element grouping is based on the following sources: D. melanogaster [32], Anastrepha species [27], C. capitata [7], and Bactrocera species. (PDF 248 kb) [file 12863_2016_342_MOESM1_ESM.pdf]

A

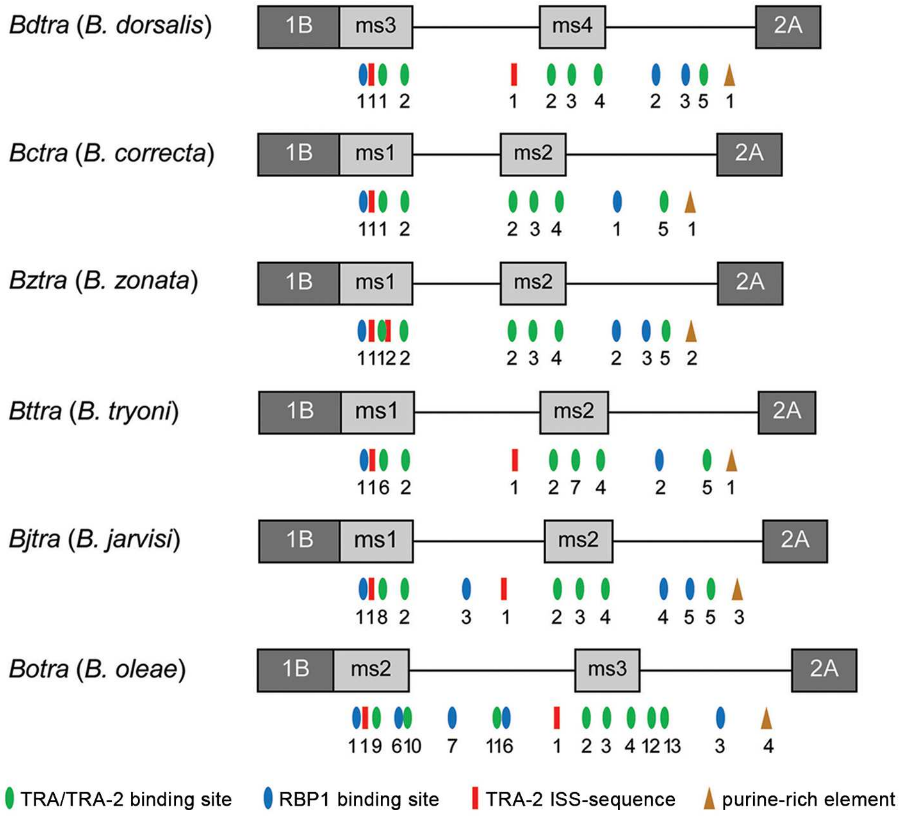

B TRA/TRA-2 binding sites

D. melanogaster	*Anastrepha* spp.	*C. capitata*	*Bactrocera* spp.
AA A<br>TC C **ATCAAC**A<br>TT G	A A CA A<br>C N **ATCAAC**<br>G T AC T<br>G	CAA CAACA<br>N **CAAT**<br>ATT TGTAG<br>T T	ACTC CAACA<br>**CAAT**<br>GTAA AGCAT<br>TA T T G

RBP1 binding sites

D. melanogaster	*Anastrepha* spp.	*C. capitata*	*Bactrocera* spp.
C<br>**ATC** NN**A**<br>T	CA<br>**ATC**T **A**<br>AT<br>T	TA<br>**ATC** N**A**<br>CG	TT<br>**ATC** N**A**<br>CA

TRA-2 ISS-sequences

D. melanogaster	*Anastrepha* spp.	*C. capitata*	*Bactrocera* spp.
G<br>**CAAG**<br>A	A<br>**CAAG**<br>G	A<br>**CAAG**<br>G	G<br>**CAAG**<br>A
