# Supplementary material for: The study of the transformer gene from Bactrocera dorsalis and B. correcta with putative core promoter regions
Source: BMC Genet. 2016 Feb 1;17:34. doi: 10.1186/s12863-016-0342-0 (PMC4736151; doi:10.1186/s12863-016-0342-0)
Supplement: Additional file 2: Table S1. — Sequence of the putative TRA/TRA-2 binding sites, RBP1 binding sites, TRA-2 ISS sequences, and purine-rich elements. (PDF 219 kb) [file 12863_2016_342_MOESM2_ESM.pdf]

Table S1 - Sequence of the putative TRA/TRA-2 binding sites, RBP1 binding sites, TRA-2 ISS sequences, and purine-rich elements

Tra/Tra-2 binding sites	RBP1 binding sites	TRA-2 ISS sequences	Purine-rich elements
1. GCACCAATCAACT	1. **ATCTTGA**	1. **CAAGG**	1. GAAAGCTGCGATGAAAAA
2. **ACTACAATCAACA**	2. ATCTTTA	2. CAAGA	2. GAAGGCTGCGATGAAAAA
3. ACAACAATCAACA	3. ATCTTAA		3. GAAAGCTGCAATGAAAAA
4. **ACAACAATCAACT**	4. ATCCTTA		4. GAAAGCTGCAATGGAAAA
5. GTAACAATTAAAT	5. ATCCTAA		
6. GCACCAATCAACA	6. ATCTACA		
7. GCATCAATCAACA	7. ATCTAAA		
8. GCACCAATCAACG			
9. ACACCAATCAACG			
10. TCTACAATCAACA			
11. TTTTCAATAAACA			
12. TATACAATAACAT			
13. ATAACAATTGAAA			

The numbers in front of each sequence correspond to the numbers for these binding sites in Additional file 1: Figure S1 (A). The conserved sequences in all *Bactrocera* species are represented in bold text.
