# Supplementary figures and images for: The study of the transformer gene from Bactrocera dorsalis and B. correcta with putative core promoter regions
Source: BMC Genet. 2016 Feb 1;17:34. doi: 10.1186/s12863-016-0342-0 (PMC4736151; doi:10.1186/s12863-016-0342-0)

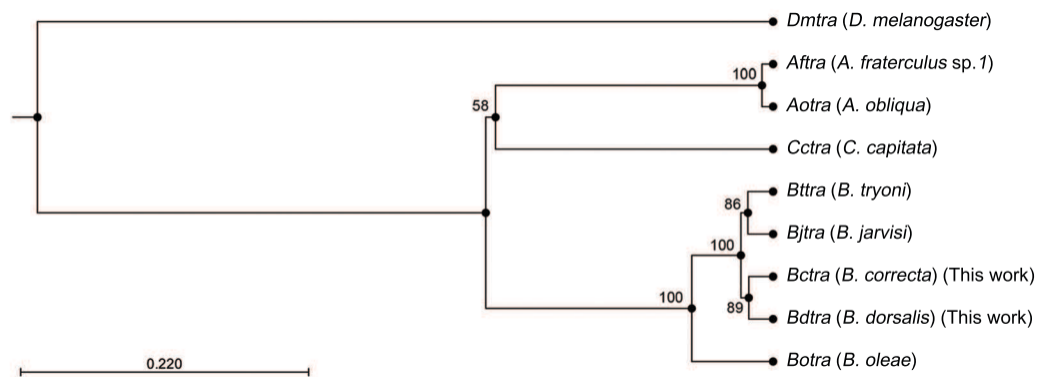

Supplement: Additional file 3: Figure S2. — Molecular phylogeny reconstructed from the female tra CDS. The tree was plotted using the UPGMA method. The horizontal branch-lengths are proportional to the genetic distance based on Jukes-Cantor measurement. The numbers shown at branch points correspond to bootstrap values from 1000 replicates with the 50 % majority rule. (PDF 821 kb) [file 12863_2016_342_MOESM3_ESM.pdf]

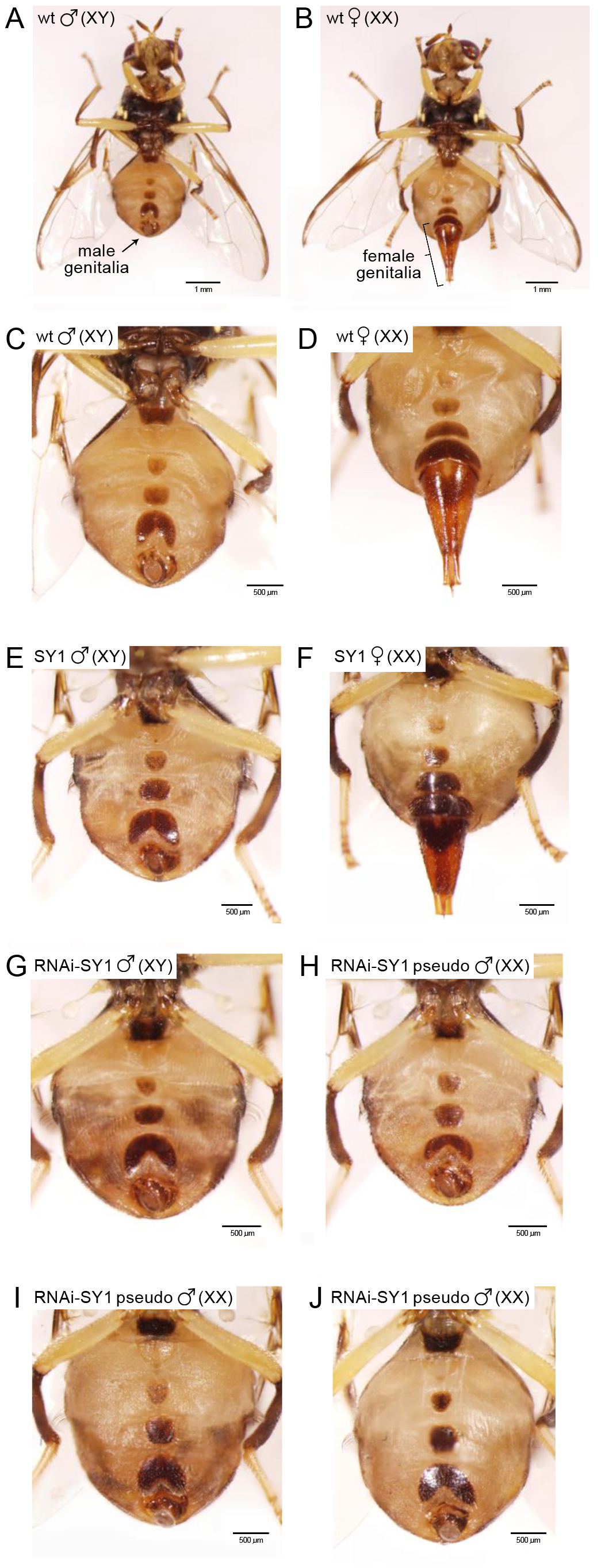

Supplement: Additional file 6: Figure S5. — Phenotypic analysis of male genitalia from tra RNAi-treated pseudomales. (A) and (B) represent the ventral view of male and female individuals, respectively, from the wild-type (wt) B. dorsalis (Phayathai1 strain) while (C) and (D) illustrate a closer look at the normal male and female genitalia, respectively. (E) and (F) are also respectively the genitalia and ovipositor from a brown-pupae male and white-pupae female of the Salaya1 (SY1), GSS. (G) is the genitalia of the tra RNAi-treated brown-pupae male whereas (H) to (J) are the genitalia of the tra RNAi-treated white-pupae pseudomale. (XX) and (XY) are presumably sexual karyotypes of each individual sub-figure. (JPG 1087 kb) [file 12863_2016_342_MOESM6_ESM.jpg]

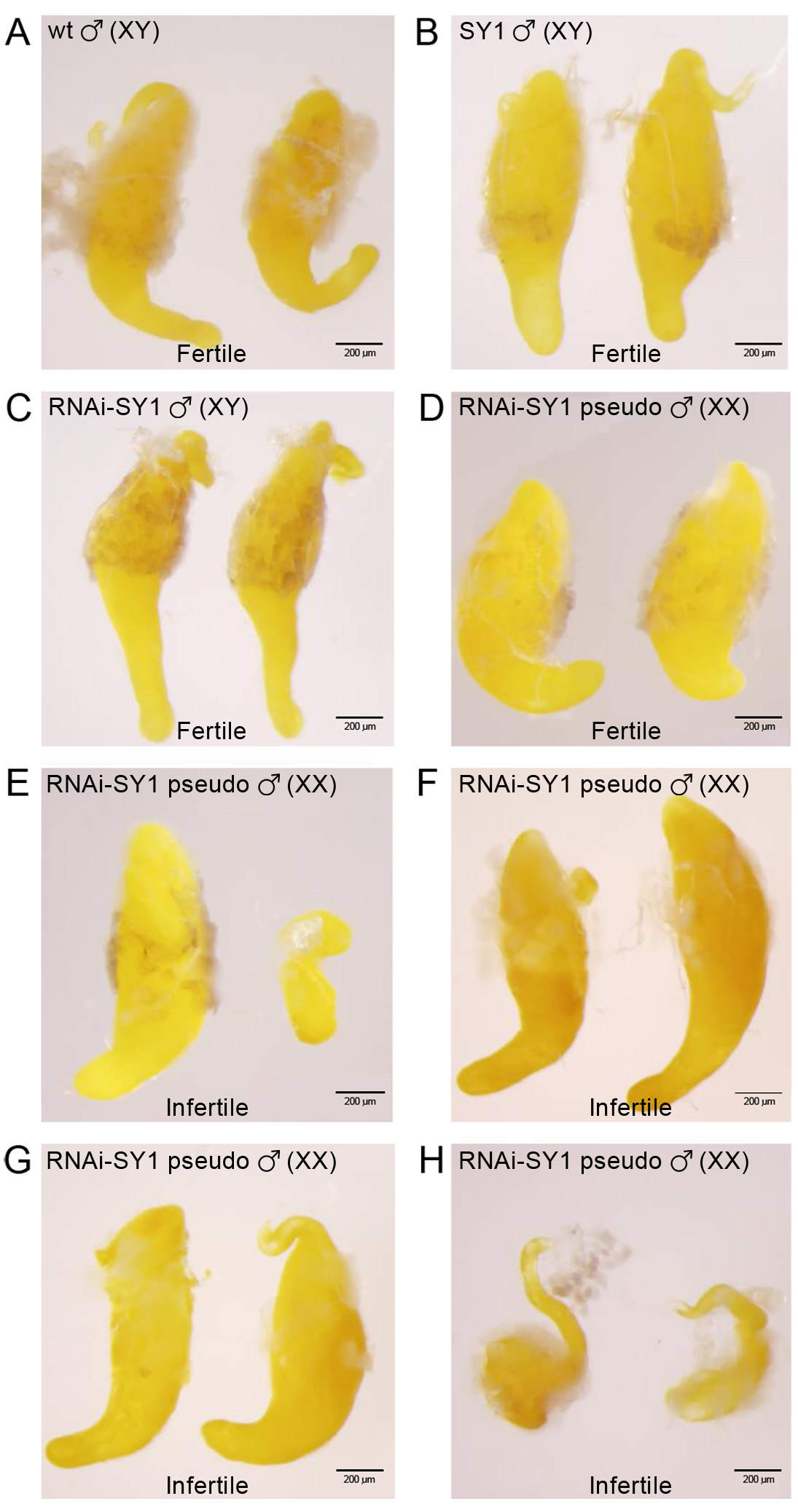

Supplement: Additional file 7: Figure S6. — The development of testes dissected from males and pseudomales. (A), (B), (C), and (D) represent a pair of fertile and fully developed testes from a wild-type (wt) male (Phayathai1 strain), a brown-pupae male (Salaya1 (SY1), GSS), a tra RNAi-treated brown-pupae male, and a tra RNAi-treated white-pupae pseudomale, respectively. (E) to (H) show a pair of sterile and aberrant testes from tra RNAi-treated white-pupae pseudomales that had no observed courtship behavior. (XX) and (XY) are presumably sexual karyotypes of each individual sub-figure. (JPG 659 kb) [file 12863_2016_342_MOESM7_ESM.jpg]
