# Supplementary material for: The study of the transformer gene from Bactrocera dorsalis and B. correcta with putative core promoter regions
Source: BMC Genet. 2016 Feb 1;17:34. doi: 10.1186/s12863-016-0342-0 (PMC4736151; doi:10.1186/s12863-016-0342-0)
Supplement: Additional file 5|: Figure S4. — TEP region of TRA protein alignment from tephritid fruit flies. The TEP region is in bold text and highlighted in pink. The identical amino acids (*) are in heavy shade. The semi-conservative amino acids (: or .) are highlighted in medium shade while the less conserved regions are in the lightest shade. (PDF 4253 kb) [file 12863_2016_342_MOESM5_ESM.pdf]

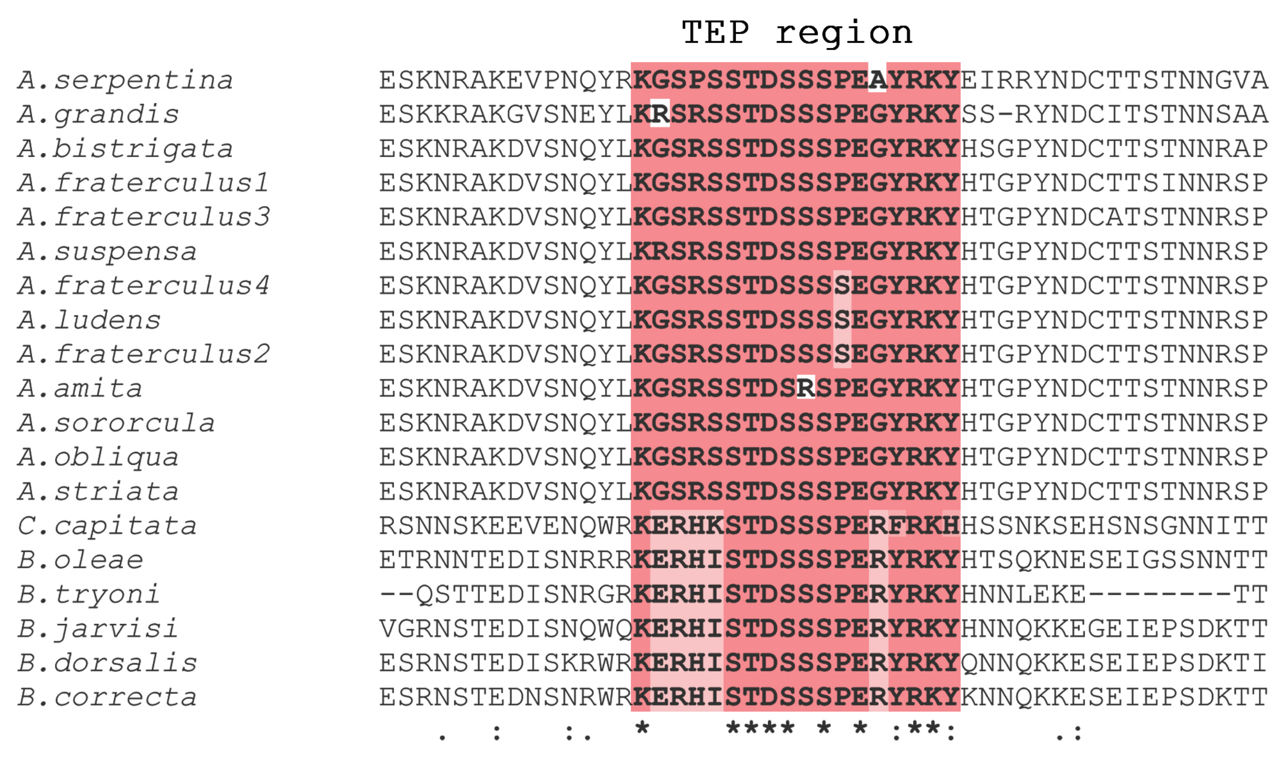
TEP region
A.serpentina ESKNRAKEVPNQYRKGSPSSTDSSSPEAYRKYEIRRYNDCTTSTNNGVA
A.grandis ESKKRAKGVSNEYLKRSRSSTDSSSPEGYRKYSS-RYNDCITSTNNSAA
A.bistrigata ESKNRAKDVSNQYLKGSRSSTDSSSPEGYRKYHSGPYNDCTTSTNNRAP
A.fraterculus1 ESKNRAKDVSNQYLKGSRSSTDSSSPEGYRKYHTGPYNDCTTSINNRSP
A.fraterculus3 ESKNRAKDVSNQYLKGSRSSTDSSSPEGYRKYHTGPYNDCATSTNNRSP
A.suspensa ESKNRAKDVSNQYLKRSRSSTDSSSPEGYRKYHTGPYNDCTTSTNNRSP
A.fraterculus4 ESKNRAKDVSNQYLKGSRSSTDSSSSEGYRKYHTGPYNDCTTSTNNRSP
A.ludens ESKNRAKDVSNQYLKGSRSSTDSSSSEGYRKYHTGPYNDCTTSTNNRSP
A.fraterculus2 ESKNRAKDVSNQYLKGSRSSTDSSSSEGYRKYHTGPYNDCTTSTNNRSP
A.amita ESKNRAKDVSNQYLKGSRSSTDSRSPEGYRKYHTGPYNDCTTSTNNRSP
A.sororcula ESKNRAKDVSNQYLKGSRSSTDSSSPEGYRKYHTGPYNDCTTSTNNRSP
A.obliqua ESKNRAKDVSNQYLKGSRSSTDSSSPEGYRKYHTGPYNDCTTSTNNRSP
A.striata ESKNRAKDVSNQYLKGSRSSTDSSSPEGYRKYHTGPYNDCTTSTNNRSP
C.capitata RSNNSKEEVENQWRKERHKSTDSSSPERFRKHHSSNKSEHSNSGNNITT
B.oleae ETRNNTEDISNRRRKERHISTDSSSPERYRKYHTSQKNESEIGSSNNTT
B.tryoni --QSTTEDISNRGRKERHISTDSSSPERYRKYHNNLEKE--------TT
B.jarvisi VGRNSTEDISNQWQKERHISTDSSSPERYRKYHNNQKKEGEIEPSDKTT
B.dorsalis ESRNSTEDISKRWRKERHISTDSSSPERYRKYQNNQKKESEIEPSDKTI
B.correcta ESRNSTEDNSNRWRKERHISTDSSSPERYRKYKNNQKKESEIEPSDKTT
. : :. * **** * * :**: .:
