# Supplementary material for: The study of the transformer gene from Bactrocera dorsalis and B. correcta with putative core promoter regions
Source: BMC Genet. 2016 Feb 1;17:34. doi: 10.1186/s12863-016-0342-0 (PMC4736151; doi:10.1186/s12863-016-0342-0)
Supplement: Additional file 8: Table S2. — GenBank Accession Numbers of transformer genes in Tephritid species and Drosophila melanogaster. (PDF 94 kb) [file 12863_2016_342_MOESM8_ESM.pdf]

Table S2 - GenBank Accession Numbers of *transformer* genes in Tephritid species and *Drosophila melanogaster*

Species	Accession Number	References
Drosophila melanogaster	NM_079390.3	-
Ceratitis capitata	AF434936.1<br>AF434937.1<br>AF434938.1	7
Anastrepha obliqua<br>*Anastrepha aff. fraterculus* 1<br>*Anastrepha aff. fraterculus* 2<br>*Anastrepha aff. fraterculus 3*<br>*Anastrepha aff. fraterculus 4*<br>*Anastrepha serpentina*<br>*Anastrepha striata*<br>*Anastrepha bistrigata*<br>*Anastrepha sororcula*<br>*Anastrepha grandis*<br>*Anastrepha amita*<br>*Anastrepha ludens*	EU024498.1<br>EU024499.1<br>EU024500.1<br>EU024501.1<br>EU024502.1<br>EU024504.1<br>EU024506.1<br>EU024507.1<br>EU024505.1<br>EU024503.1<br>EU024508.1<br>EU024509.1	27
Anastrepha suspensa	JN597286.1	59
Bactrocera dorsalis	KU254107<br>KU254111<br>KU254112<br>KU254113<br>KU254114<br>KU254115<br>KU254118	This work
Bactrocera correcta	KU254106<br>KU254116<br>KU254117<br>KU254119	This work
Bactrocera zonata	KU254108	This work
Bactrocera carambolae	KU254109	This work
Bactrocera tryoni	KU254110	This work

Table S2 - GenBank Accession Numbers of *transformer* genes in Tephritid species and *Drosophila melanogaster* (continued)

Species	Accession Number	References
Bactrocera jarvisi	KJ443716.1<br>KJ443718.1<br>KJ443721.1	8
Bactrocera tryoni	KJ443717.1<br>KJ443719.1<br>KJ443720.1	8
Bactrocera oleae	DQ100251.1<br>DQ100252.1<br>AJ715413.1<br>AJ715414.1	26
Bactrocera dorsalis	KP342058.1<br>KP342059.1<br>KP342060.1	28
	KP769559.1<br>KP769560.1<br>KP769561.1	29
Bactrocera zonata	KJ397268.1	61
