# Supplementary material for: The study of the transformer gene from Bactrocera dorsalis and B. correcta with putative core promoter regions
Source: BMC Genet. 2016 Feb 1;17:34. doi: 10.1186/s12863-016-0342-0 (PMC4736151; doi:10.1186/s12863-016-0342-0)
Supplement: Additional file 9: Table S3. — GenBank Accession Numbers of doublesex genes in Tephritid species and Drosophila melanogaster. (PDF 87 kb) [file 12863_2016_342_MOESM9_ESM.pdf]

Table S3 - GenBank Accession Numbers of *doublesex* genes in Tephritid species and *Drosophila melanogaster*

Species	Accession Number	References
Drosophila melanogaster	NM_169203.2	-
Ceratitis capitata	AF435087.2	Unpublished
Anastrepha obliqua	AY948420.1	41
Anastrepha aff. fraterculus 1	DQ494344.1	Unpublished
Bactrocera jarvisi	KJ816788.1	8
Bactrocera oleae	AJ547621.1	40
Bactrocera tryoni	AF029675.1	39
Bactrocera dorsalis	FJ176944.1	44
Bactrocera correcta	FJ185166.1	44
