# Supplementary material for: The study of the transformer gene from Bactrocera dorsalis and B. correcta with putative core promoter regions
Source: BMC Genet. 2016 Feb 1;17:34. doi: 10.1186/s12863-016-0342-0 (PMC4736151; doi:10.1186/s12863-016-0342-0)
Supplement: Additional file 10: Table S4. — Sequence of primers used in this study. (PDF 78 kb) [file 12863_2016_342_MOESM10_ESM.pdf]

Table S4 - Sequence of primers used in this study

**Primers**	**Sequence (5' to 3')**	**References**
Oligo (dT) adapter	CGGGACTCGTCGACATCGAT(17)	39
Adapter	CGGGACTCGTCGACATCG	
***transformer* gene**		Designed in this study
BoF	TGCAGTTCGACGTGACGTAT	
BoR	GGTTCCTGCGGATTTTATGA	
5U-BD1	CTTAACCAGACATTTAGATAACGG	
5U-BD1_R	CCGTTATCTAAATGTCTGGTTAAG	
5U-BC1	TCAGACATTTAGRTAACGGCCCT	
5U-BC1_R	AGGGCCGTTAYCTAAATGTCTGA	
5U-BD3	CAAAGTAATCGCTGTGATGAGTTA	
5U-BD3_R	TAACTCATCACAGCGATTACTTTG	
5U-BC4	ATGCGATGAGTATTTTTGTATGCG	
5U-BC4_R	CGCATACAAAAATACTCATCGCAT	
1B-F	GAAGTTGTTATTAAGCGTAGATTCGG	
1B-TraF	AAGGTCCTCACGCTATAGAACG	
1B-1R	CCGAATCTACGCTTAATAACAACTTC	
ms-F	AATGACGCACCAATCAACTTACC	
ms-R	GGAATGTTGATTGTTGTTTGTGTTGC	
2A-2R	CTGTGCTGTTTCTACTTTCAGTTTG	
2B-F	ACCAAGTAAGGGAGCGAGACAGAA	
2B-R2	TTCGCCATTCCCATTCTGTTCTTC	
2B-R1	CTTTCCCGTTCGCGTTTACTATTG	
Nested TraF	GACGCAGGCGTAGTATTTCAAGGT	
3Tra-R	CAATGGTGGTARAACAGCAGGCA	

Table S4 - Sequence of primers used in this study (continued)

Primers	Sequence (5' to 3')	References
***B. correcta tra* gene**		
BcT-3_*Nco*I	ACAGTCCATGGTATCTCCAAAACCATAAGTTAGCGG	Designed in this study
BcT-4_*Xba*I	AGCATTCTAGACATGTTGAAGGCTGCCTTAAATGAT	
**DsRed fragment**		
dsRed_*Xba*IF	CGGTCTCTAGAATGGTGCGCTCCTCCAAG	Designed in this study
dsRed_*BamH*IR	ATGATGGATCCTCGCGGCCGCTACAGGAACAGG	
**LOC105232903 gene**		Designed in this study
Tpro	CTATCGCTTGGTGGACCGCAG	
***doublesex* gene**		
C3	GCTTAACCGCCGATCGTCAG	44
Female-specific (*f*)	GTATTCGTTTACGACATGTTGGC	
C4	GCTGGGGCAGATATTGAAGAG	
Male-specific (*m*)	CGGTACTAAGGGTTTAGTCATC	
