# Supplementary material for: The study of the transformer gene from Bactrocera dorsalis and B. correcta with putative core promoter regions
Source: BMC Genet. 2016 Feb 1;17:34. doi: 10.1186/s12863-016-0342-0 (PMC4736151; doi:10.1186/s12863-016-0342-0)
Supplement: Additional file 4: Figure S3. — Aligned sequences of putative TRA polypeptides of the Anastrepha species. The consensus sequences of Anastrepha species are represented in bold text above each line, and the points stand for the same amino acids. The GenBank Accession Numbers of Anastrepha tra genes were consolidated in Additional file 8: Table S2. (PDF 458 kb) [file 12863_2016_342_MOESM4_ESM.pdf]

```
Consensus         MNIPKASTATRKIQIEQSVPTGSIRKGPYAIERSVDASEVVIKRRFGEGSKPLFQRDDIVVNPNSVIKAADSHTEKHLISESKNR
A.suspensa        .S..................A........................................K.R......E.....Y........
A.striata         .....................................................................................
A.fraterculus1    ..............................................................................L......
A.sororcula       .S...................................................................T...............
A.obliqua         .S...................................................................T...............
A.fraterculus3    .S...................................................................T...............
A.ludens          .S...................................................................................
A.fraterculus2    .S.............................................................D.....................
A.fraterculus4    ..V...........................................................S............N.........
A.amita           ..................I..................N...............................................
A.bistrigata      ............................H........N...............................G...............
A.grandis         ..................I..................N.............................NV..............K.
A.serpentina      .....................................N.............................N.................

Consensus         AKDVSNQYLKGSRSSTDSSSPEGYRKYHTGPYNDCTTSTNNRSPPRTKPLKSTNEGKHTIRCDSSPPFNHRRRTPEKLPYFIDET
A.suspensa        ..........R..........................................................................
A.striata         .................................................................................V...
A.fraterculus1    ......................................I...............................H..........V...
A.sororcula       .............................................................S.......................
A.obliqua         .............................................................S.......................
A.fraterculus3    ...................................A..........P..............S.....................T.
A.ludens          ....................S....................................................P...........
A.fraterculus2    ....................S.......................................H........................
A.fraterculus4    ....................S................................................................
A.amita           ..................R..................................................................
A.bistrigata      ............................S.............A..........S...............................
A.grandis         ..G...E...R................SS-R..........SAA...........R.R......P....................
A.serpentina      ..E.P...R...P.........A....EIRR..........GVAT....F.......R.F.D.......................

Consensus         RERDRLRRKYETKPKIPSSSPPLSSRSRHRRSRSKSRSRSQSREFEHRLKYANLRRSVSTDRYIGETGRRDRGSRSDKTSSRYRH
A.suspensa        ...........RS....................T...................................................
A.striata         .............S.......................................................................
A.fraterculus1    .............S...............................D.......................................
A.sororcula       ................L...........................L.........................E..............
A.obliqua         ..........................P.................L.........................E..............
A.fraterculus3    ..........................P.................L........P...............................
A.ludens          .........................................................I...........................
A.fraterculus2    .....................................................................................
A.fraterculus4    .Q........................P.....H....................................................
A.amita           .......................................................................G.............
A.bistrigata      ........................................H.........H...................E......R.......
A.grandis         ..................T....T...........P....H.........P..F.............S..E.....E........
A.serpentina      ..........DI.....P..................W...H.........GD.R.........T......E.R............

Consensus         RSEDRSKSDRRQRHWRSPSRSPRRSRDRLTRSRKQSRHDERHKNYHSEHGGASSDELAQRNLPQPQIITIPVPVPADFMNYTYPT
A.suspensa        .....................................Y..................T............................
A.striata         .....................................................................................
A.fraterculus1    .......................................K.............................................
A.sororcula       .....................................................................................
A.obliqua         .....................................................................................
A.fraterculus3    .....................................................................................
A.ludens          ...N......................................M..........................................
A.fraterculus2    .......N................................G.M..........................................
A.fraterculus4    ..........................................MD.Y.......................................
A.amita           ..Q..............S........................M...............................L..........
A.bistrigata      .......N..............S..............................T...............................
A.grandis         .......N..............S.........H...........C....K.S.N.........P.....................
A.serpentina      .......N..............S.L..........................T.N........................I......

Consensus         WPTQWNPPMAHPVRYGPPAAYHIPTILPAAVMPPMRPPLPPYGPHPPPLRYGVRGFRLPPQYGASRPWRPNFRTKNT
A.suspensa        .............................................................................
A.striata         ......S......................................................................
A.fraterculus1    .............................................................................
A.sororcula       ..A..........................................................................
A.obliqua         .............................................................................
A.fraterculus3    ....................................................G........................
A.ludens          .............................................................................
A.fraterculus2    .............................................................................
A.fraterculus4    ..............................................S............................K.
A.amita           ..........................................D..................................
A.bistrigata      ..............................................T..............................
A.grandis         ..............................................................N..............
A.serpentina      ...................T............................V............................
```
